# Dendrons containing boric acid and 1,3,5-tris(2-hydroxyethyl)isocyanurate covalently attached to silica-coated magnetite for the expeditious synthesis of Hantzsch esters

**DOI:** 10.1038/s41598-020-80884-z

**Published:** 2021-01-27

**Authors:** Mahsa Sam, Mohammad G. Dekamin, Zahra Alirezvani

**Affiliations:** Pharmaceutical and Heterocyclic Compounds Research Laboratory, Department of Chemistry, Iran University of Science and Technology, 1684613114 Tehran, Iran

**Keywords:** Environmental sciences, Chemistry, Materials science, Nanoscience and technology

## Abstract

A new multifunctional dendritic nanocatalyst containing boric acid and 1,3,5-tris(2-hydroxyethyl)isocyanurate covalently attached to core–shell silica-coated magnetite (Fe_3_O_4_@SiO_2_@PTS-THEIC-(CH_2_)_3_OB(OH)_2_) was designed and properly characterized by different spectroscopic or microscopic methods as well as analytical techniques used for mesoporous materials. It was found that the combination of both aromatic π–π stacking and boron–oxygen ligand interactions affords supramolecular arrays of dendrons. Furthermore, the use of boric acid makes this dendritic catalyst a good choice, from corrosion, recyclability and cost points of view. The catalytic activity of Fe_3_O_4_@SiO_2_@PTS-THEIC-(CH_2_)_3_OB(OH)_2_, as an efficient magnetically recoverable catalyst, was investigated for the synthesis of polyhydroacridines (PHAs) as well as polyhydroquinolines (PHQs) via one-pot multicomponent reactions of dimedone and/or ethyl acetoacetate, different aldehydes and ammonium acetate in EtOH under reflux conditions. Very low loading of the catalyst, high to quantitative yields of the desired PHAs or PHQs products, short reaction times, wide scope of the substrates, eliminating any toxic heavy metals or corrosive reagents for the modification of the catalyst, and simple work-up procedure are remarkable advantages of this green protocol. An additional advantage of this magnetic nanoparticles catalyst is its ability to be separated and recycled easily from the reaction mixture with minimal efforts in six subsequent runs without significant loss of its catalytic activity. This magnetic and dendritic catalyst can be extended to new two- and three-dimensional covalent organic frameworks with different applications.

## Introduction

New materials are required to be developed for the modern science and technology. These new materials are used for different applications such as drug delivery, medical diagnosis, reinforced composites, semiconductors, electronics, optics, sensors, sorbents, CO_2_ capture, heterogeneous catalysis, etc. In this manner, nanomaterials can play a vital role^1–9^. One of the emerging fields for the preparation and fabrication of new nanomaterials is dendrimer chemistry which has been recently expanded as two- or three-dimensional covalent organic frameworks (COFs). These strategies afford multifunctional materials which demonstrate synergistic effects and hence, higher performance and efficacy as well as newer and more specific properties than previous counterparts^1,10–24^. In addition, dendrimers can encapsulate and consequently, stabilize metallic catalytic active nanoparticles^25–28^. Furthermore, the properties of new materials can be modified and improved by their immobilization onto the surface of magnetic nanoparticles (MNPs), especially in the case of heterogeneous catalysis^26,29–36^. These improvements include better separation using an external magnetic field^34,35,37–40^, enhancement of the reaction rates by MNPs via local heating through induction and increasing the surface area as well as synergistic effects in conjunction with other catalytic species or centers due to the catalytic performance of magnetic materials, including Fe, Ni, Co or Ce-based ones^41–43^. Hence, active catalytic species or centres supported onto the surface of MNPs have received much attention in the field of heterogeneous catalysis for promoting organic reactions in recent years^26,44–46^.

As a particular type of magnetic nanoparticles, superparamagnetic iron oxide nanoparticles (SPIONs) are more widely available than other MNPs due to advantages such as biologically well-accepted constituents, established size-selective preparation, diminished agglomeration, ease of preparation, and lower cost^26,45,47–57^. On the other hand, heterogenization of the active sites of usual dendritic catalysis has been pursued by either attaching the catalyst covalently within the dendrimer core or at the branch termini as well as through supramolecular interactions such as metal–ligand, hydrogen bonding, aromatic π–π stacking, hydrophobic and van der Waals forces^22,26,51,58–61^. Therefore, design and preparation of new magnetic dendritic catalytic systems by appropriate application of dendron segments which can be expanded to 2D or 3D covalent organic frameworks (COFs) is still in high demand.

**Scheme 1:**
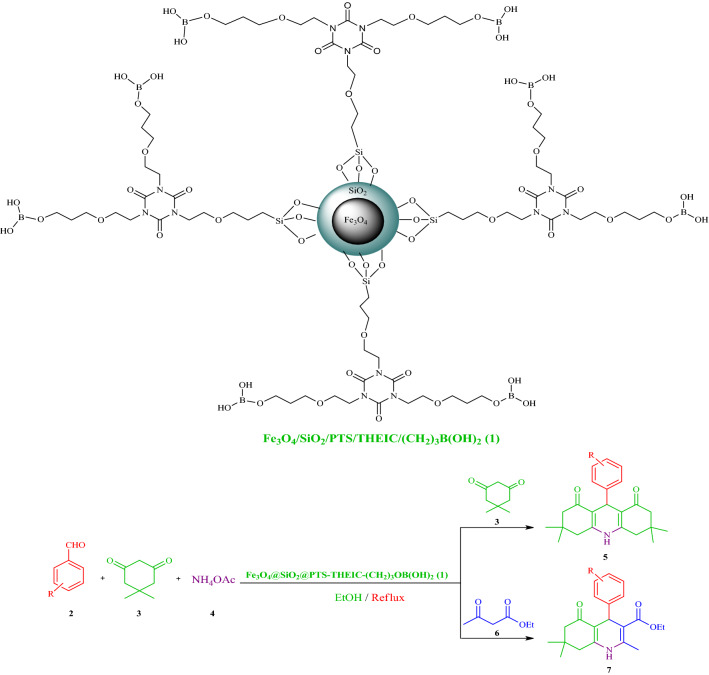
Schematic representation of the Fe_3_O_4_@SiO_2_@PTS-THEIC-(CH_2_)_3_OB(OH)_2_ catalyst (**1**) and its catalytic activity in the one-pot synthesis of polyhydroacridines (**5**) and polyhydroquinolines (**7**) through multicomponent reaction (MCR) strategy (Drawn using the ChemDraw Ultra 12.0 software developed by PerkinElmer).

In recent years, thermally stable heteroaromatic 1,3,5-triazinane-2,4,6‐trione (isocyanurate) moiety has received significant attention in polymer and material chemistry due to its numerous industrial applications, particularly in the field of low toxic drug-delivery agents, tensioactive building blocks and nonlinear optical properties, foams, surface coatings, films, paints, fibers, selective anion receptors and preparation of periodic mesoporous organosilica^1,19,62–77^. On the other hand, boric acid and its derivatives have achieved specific attention, as appropriate catalysts, in organic synthesis due to their advantages including high solubility in water, easy handling, low prices, and environmentally friendly and commercial availability^78–87^. In an attempt to indicate how applying SPIONs would affect the dendrimer bearing tridentate and thermally stable isocyanurate moiety as well as boric acid catalytic activity, this study reports the use of multifunctional dendritic nanocatalyst containing boric acid and 1,3,5-tris(2-hydroxyethyl)isocyanurate covalently attached to core–shell silica-coated SPIONs (Fe_3_O_4_@SiO_2_@PTS-THEIC-(CH_2_)_3_OB(OH)_2_, **1**), as a novel and efficient supramolecular heterogeneous catalyst, in the one-pot synthesis of polyhydroacridines (PHAs, **5**) and polyhydroquinolines (PHQs, **7**) through multicomponent reaction (MCR) strategy (Scheme 1).

MCRs are one-pot reactions that involve more than two substrates demonstrating convergence as well as very high atom efficiency and bond-forming-index (BFI)^88–90^. Thus, MCRs are usually a good alternative for the sequential multistep synthesis, especially for useful heterocyclic scaffolds such as Hantzsch esters including 1,4-dihydropyridines (DHPs), PHQs and PHAs in organic synthesis and medicinal chemistry^91–96^. Generally known as one of the main groups of nitrogen heterocycles, polyhydroquinolines (PHQs) and polyhydroacridines (PHAs) have become considerably interesting due to their significant therapeutic and pharmacological properties^97–100^. Indeed, they are used as antimalaria, calcium β-blocker, antioxidant, antimicrobial, antifungal, vasodilator, anticancer, bronchodilator, antiatherosclerotic, geroprotective, hepatoprotective and antidiabetic agents as well as in the production of laser colors, radical reservoirs and safe hydrogen transfer agents^4,101–111^. First introduced by Arthur Hantzsch in 1882, Hantzsch reaction is an MCR that contains the combination of a β-dicarbonyl compound, an aldehyde and a source consisting of ammonia (usually NH_4_OAc)^112^. However, catalytic systems are required to accelerate this multicomponent reaction. Here are some recent reported catalysts in this area: Mn@PMO-IL^103^, vanadium ion doped titania nanoparticles^113^, Lewis acidic mesoporous material (TUD-1) containing Fe^114^, magnetite nanoparticle-supported ceria^41^, silica-coated magnetic nanoparticles with tags of ionic liquid^115^, Boehmite silica sulfuric acid (Boehmite-SSA) ^116^, PMO-ICSPrSO_3_H^117^, Fe_3_O_4_@B-MCM-41^118^, PS/PTSA^119^, PdRuNi@GO^13^, 1,3,5-tris(2-hydroxyethyl) isocyanurate covalently functionalized MCM-41^120^, alginic acid^121,122^ and glycine nitrate (GlyNO_3_) ionic liquid^123^.

## Results and discussion

### Characterization of dendritic nanocatalyst containing boric acid and 1,3,5-tris(2-hydroxyethyl)isocyanurate covalently attached to core–shell silica-coated magnetite (Fe_3_O_4_@SiO_2_@PTS-THEIC-(CH_2_)_3_OB(OH)_2_, 1)

**Figure 1:**
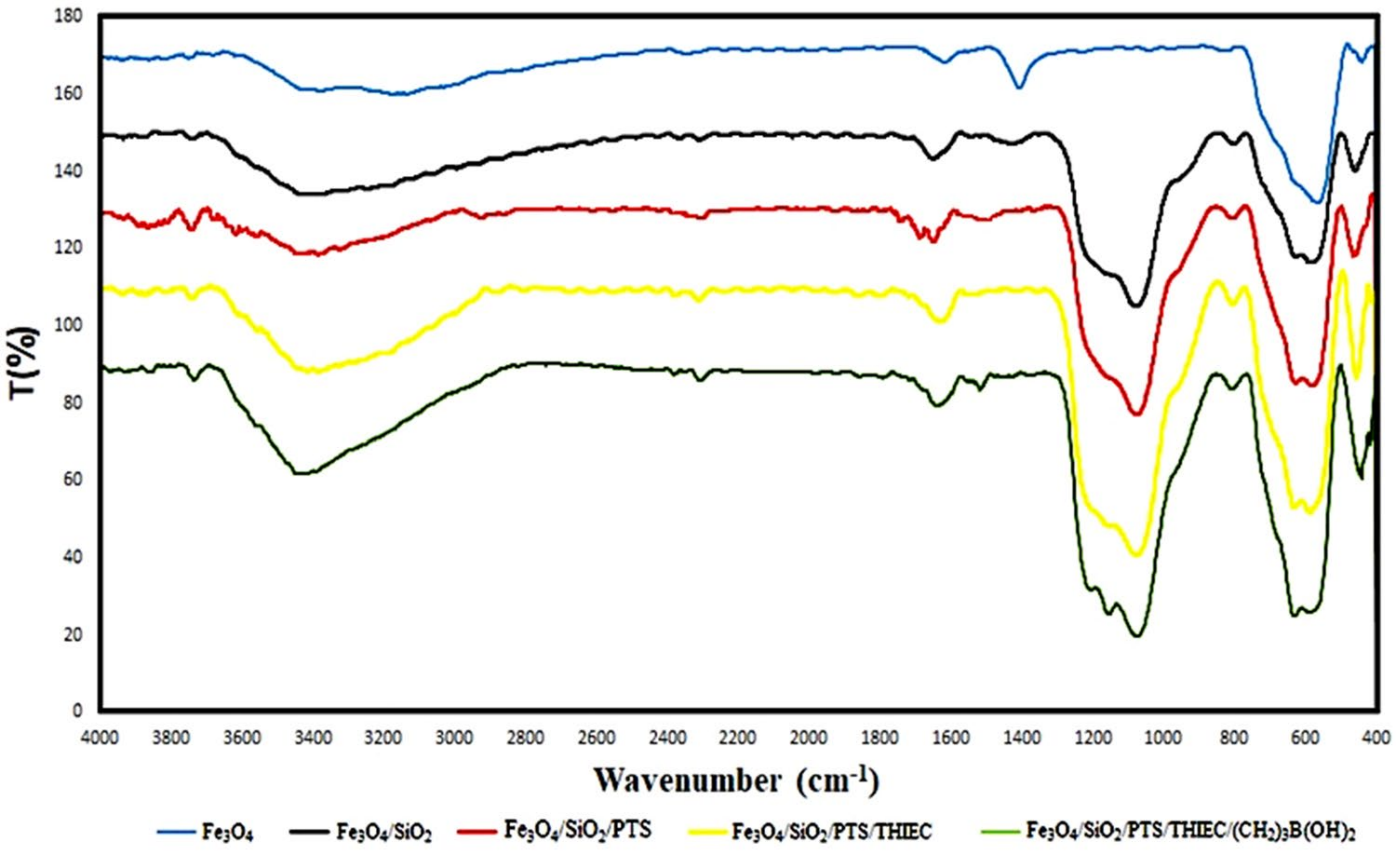
The FTIR spectra of Fe_3_O_4_, Fe_3_O_4_@SiO_2_, Fe_3_O_4_@SiO_2_@PTS, Fe_3_O_4_@SiO_2_@PTS-THEIC, Fe_3_O_4_@SiO_2_@PTS-THEIC-(CH_2_)_3_OB(OH)_2_ (**1**, from top to down, reproduced using the Microsoft Excel 2016).

At first, the boric-acid-functionalized-1,3,5-tris(2-hydroxyethyl)isocyanurate attached to the silica-coated SPIONs (Fe_3_O_4_@SiO_2_@PTS-THEIC-(CH_2_)_3_OB(OH)_2_, **1**) was characterized using different spectroscopic or analytical methods. As it has been shown in FT-IR spectrum (Fig. 1), the absorption bands at around 632 and 572 cm^−1^ are related to the Fe–O bond vibrations. On the other hand, absorption band of Si–O–Si asymmetric stretching vibrations are apparent at around 1076 cm^−1^. Furthermore, the observed signals at 954, 802 and 459 cm^–1^ are assigned to the symmetric stretching and bending vibrations of Si–O–Si bond^43,57,124^. Also, the absorption band of C=O bond vibrations of the isocyanurate moiety appeared at around 1637 cm^−1^^77,120,125^. Furthermore, the signals in range of 1350–1000 cm^−1^ belong to the C–N bonds vibrations. On the other hand, the absorption band of B–O vibrations appeared at 1510 cm^−1^. Furthermore, there is an absorption signal at around 1191 cm^−1^ which is related to B–O–H bond vibrations. Also, the signal at 563 cm^−1^ is assigned to O–B–O bond vibrations. It is generally accepted that the broad band centred at 3400 cm^−1^ is ascribed to the stretching vibrations of O–H bonds^126,127^. All of these data demonstrate that the catalyst **1** has been successfully prepared.

**Figure 2:**
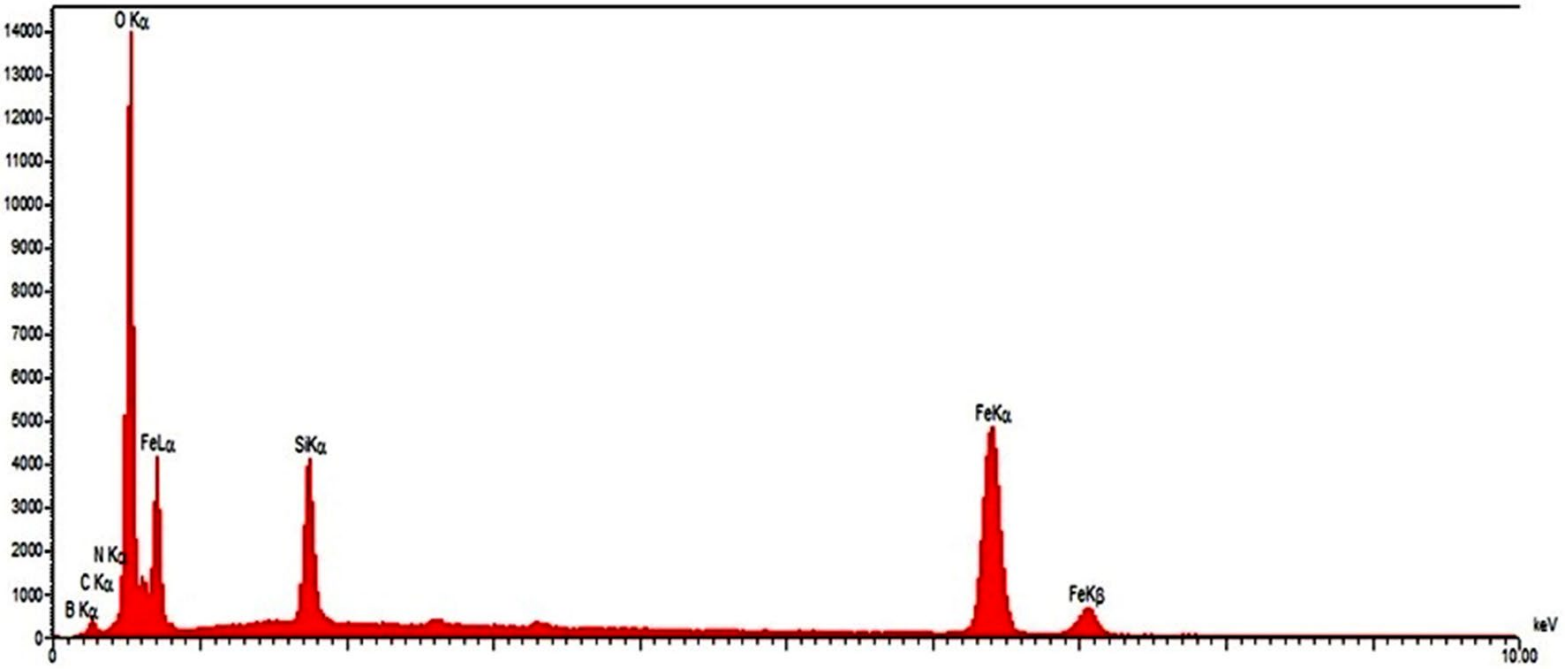
Energy dispersive spectroscopy (EDX) analysis of the magnetic Fe_3_O_4_@SiO_2_@PTS-THEIC-(CH_2_)_3_OB(OH)_2_ catalyst (**1**).

Energy dispersive spectroscopy (EDX) spectrum of Fe_3_O_4_@SiO_2_@PTS-THEIC-(CH_2_)_3_OB(OH)_2_ (**1**) proved that the magnetic catalyst functionalized with dendrons containing 1,3,5-tris(2-hydroxyethyl)isocyanurate and boric acid has been functionalized properly due to the presence of Fe, Si, O, C, N and B elements. The percentages of elements were measured to be B (1.96), C (6.99), N (2.50), O (63.58), Si (12.33) and Fe (12.65), respectively. It can be deduced from the absence of Cl and Br elements that terminal chloride groups of the 3-chloropropyl trimethoxysilane (3-APTS) linker as well as terminal bromide groups of the 1,3-dibromopropane linker have been completely replaced by covalent bonding (Fig. 2).

**Figure 3:**
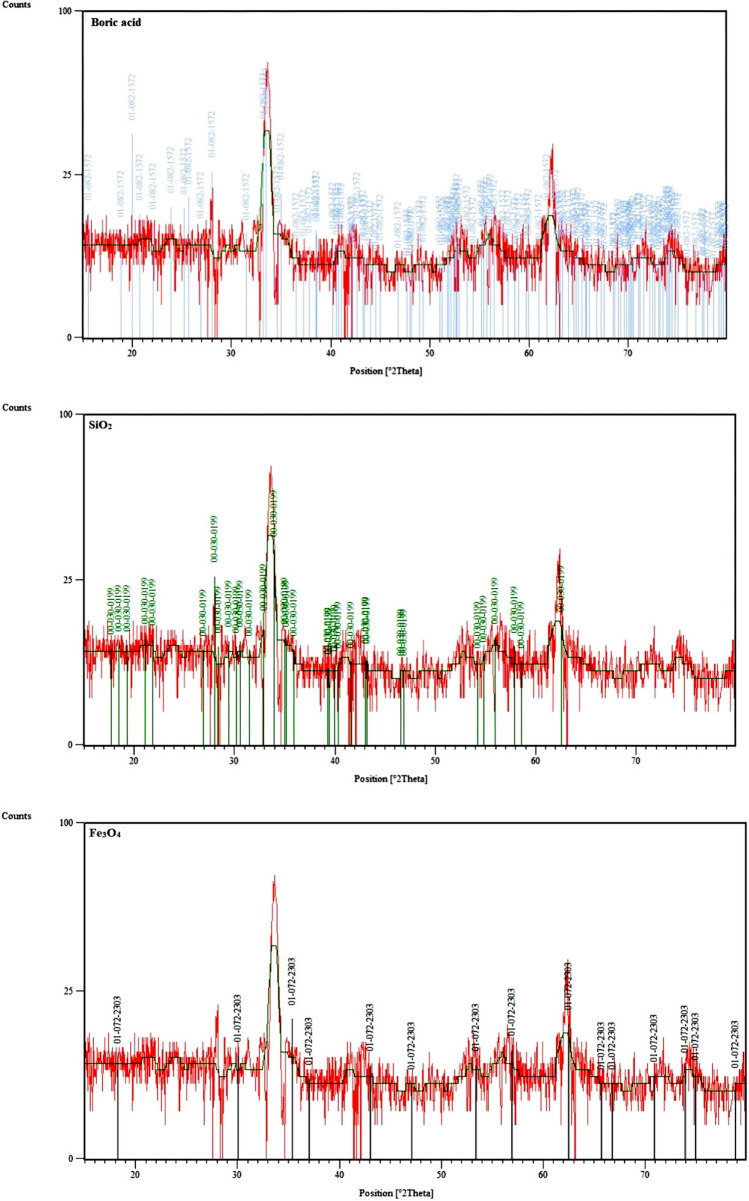
X-ray diffraction (XRD) pattern of the magnetic dendritic Fe_3_O_4_@SiO_2_@PTS-THEIC-(CH_2_)_3_OB(OH)_2_ catalyst (**1**, the individual reference card numbers of the catalyst **1** components were collected from the X'pert HighScore Plus version 2.1 software developed by the PANalytical B.V.).

The X-ray diffraction (XRD) pattern of Fe_3_O_4_@SiO_2_@PTS-THEIC-(CH_2_)_3_OB(OH)_2_ (**1**) exhibited the phase structure and crystallization of the magnetic nanomaterials (Fig. 3). The main peaks were observed at 2θ: 27.9°, 32.5°, 33.8°, 55.6°, 56.4°, 62.3°. By comparing the XRD pattern of the prepared nanocatalyst (**1**) with the reference card numbers in the X'pert software, the crystal network of Fe_3_O_4_, SiO_2_ and B(OH)_3_ correspond with 072–2303, 082–1572 and 030–0199 card numbers, respectively.

**Figure 4:**
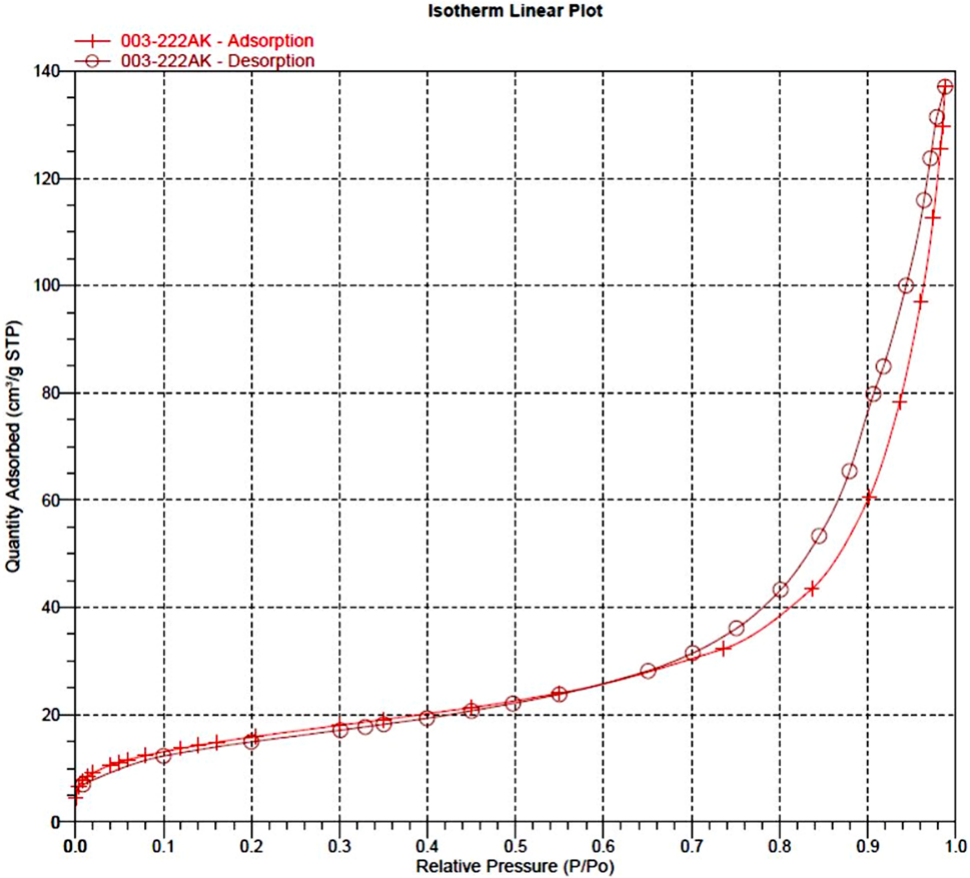
Nitrogen adsorption–desorption isotherm (BET) of the magnetic Fe_3_O_4_@SiO_2_@PTS-THEIC-(CH_2_)_3_OB(OH)_2_ catalyst (**1**).

The textural properties of the magnetic dendritic Fe_3_O_4_@SiO_2_@PTS-THEIC-(CH_2_)_3_OB(OH)_2_ catalyst (**1**) was investigated by nitrogen adsorption–desorption isotherms (Fig. 4). The BET isotherm of the prepared catalyst corresponds with the BET standard type II adsorption isotherm. The surface area (BET), pore size and pore volume of the catalyst were calculated 55.8 m^2^/g, 13.9 nm, 0.19 cm^3^/g, respectively.

**Figure 5:**
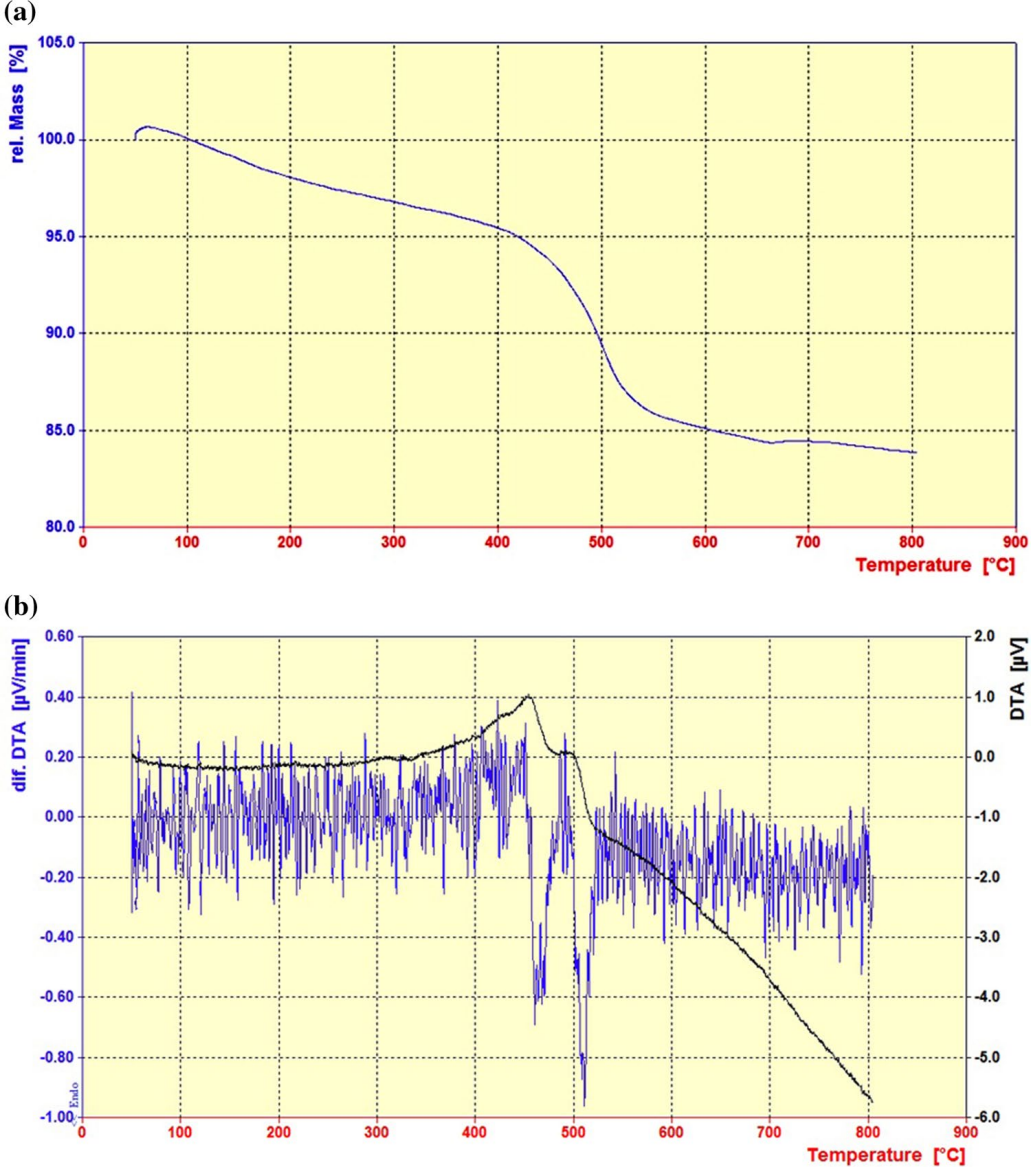
(**a**) Thermal gravimetric analysis (TGA) and (**b**) differential thermal analysis (DTA) curves of the magnetic dendritic Fe_3_O_4_@SiO_2_@PTS-THEIC-(CH_2_)_3_OB(OH)_2_ catalyst (**1**).

Thermal gravimetric analysis (TGA) and differential thermal analysis (DTA) measurements were carried out under air atmosphere by heating the sample at the rate of 10 °C min^−1^ up to 800 °C (Fig. 5). The first weight loss under 100 °C is related to the removal of water and organic solvents which have remained in the dendritic catalyst through its preparation processes. On the other hand, the second weight loss about 150 °C can be assigned to the dehydration of boric acid moieties and their condensation. Furthermore, two distinct weight losses about 460 and 510 °C are attributed respectively to the decomposition of aliphatic linkers and 1,3,5-tris(2-hydroxyethyl) isocyanurate moieties in the structure of the dendritic Fe_3_O_4_@SiO_2_@PTS-THEIC-(CH_2_)_3_OB(OH)_2_ catalyst (**1**) according to the data obtained by DTA (Fig. 5b).

**Figure 6:**
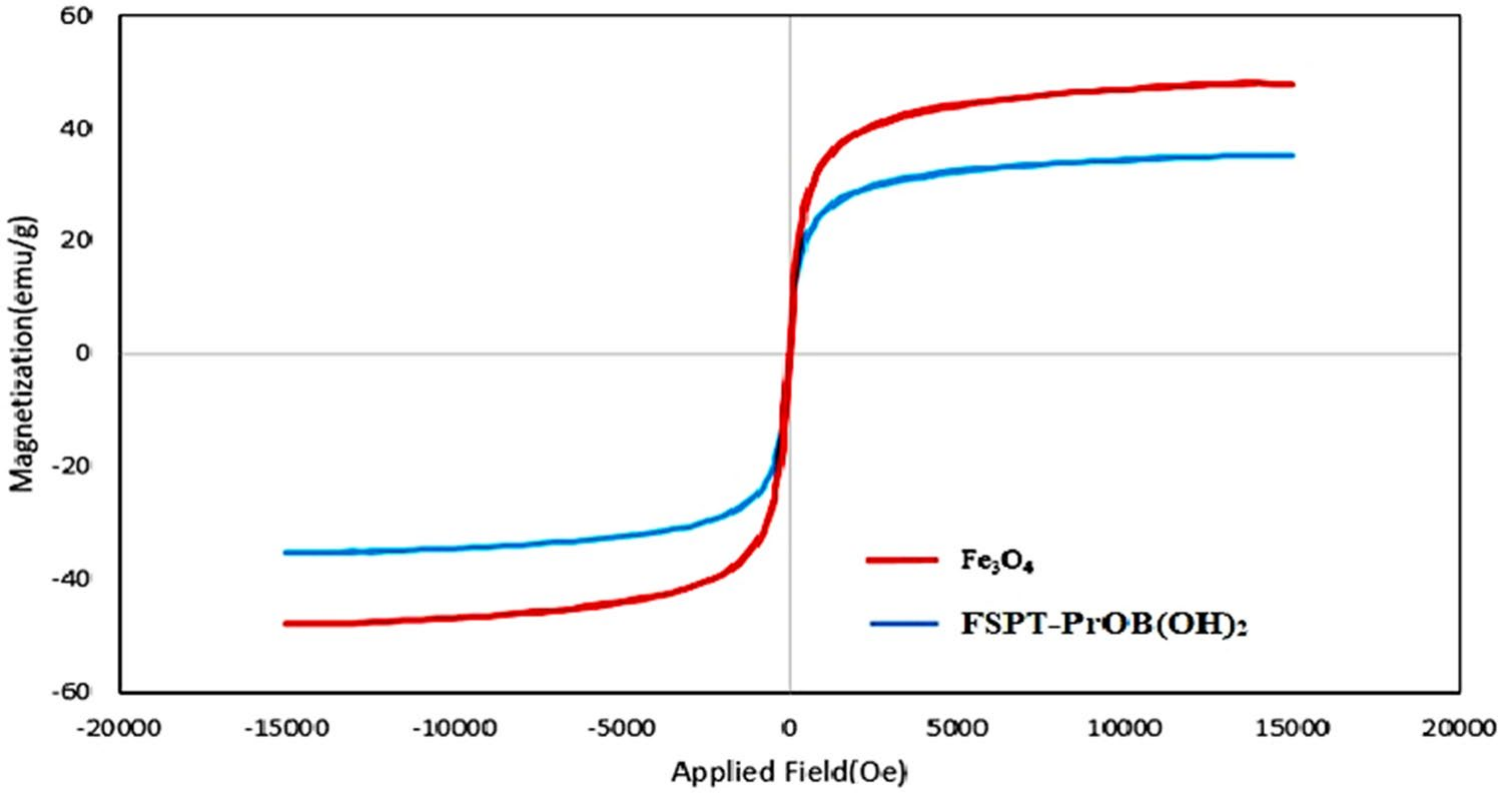
VSM analysis of the magnetic dendritic Fe_3_O_4_@SiO_2_@PTS-THEIC-(CH_2_)_3_OB(OH)_2_ catalyst (**1**, reproduced using the Microsoft Excel 2016).

Vibrating sample magnetometry (VSM) technique was used for measuring the magnetic properties of catalyst (**1**) at room temperature (Fig. 6). The saturation value of magnetization of Fe_3_O_4_ and Fe_3_O_4_@SiO_2_@PTS-THEIC-(CH_2_)_3_OB(OH)_2_ was measured to be 47.9 and 35.2 emu/g, respectively. Indeed, the reduction of saturation magnetization of Fe_3_O_4_@SiO_2_@PTS-THEIC-(CH_2_)_3_OB(OH)_2_ shows that the dendritic catalyst has been formed. However, the observed saturation magnetization of catalyst (**1**) is enough and hence, it can be easily separated by an external magnetic field.

**Figure 7:**
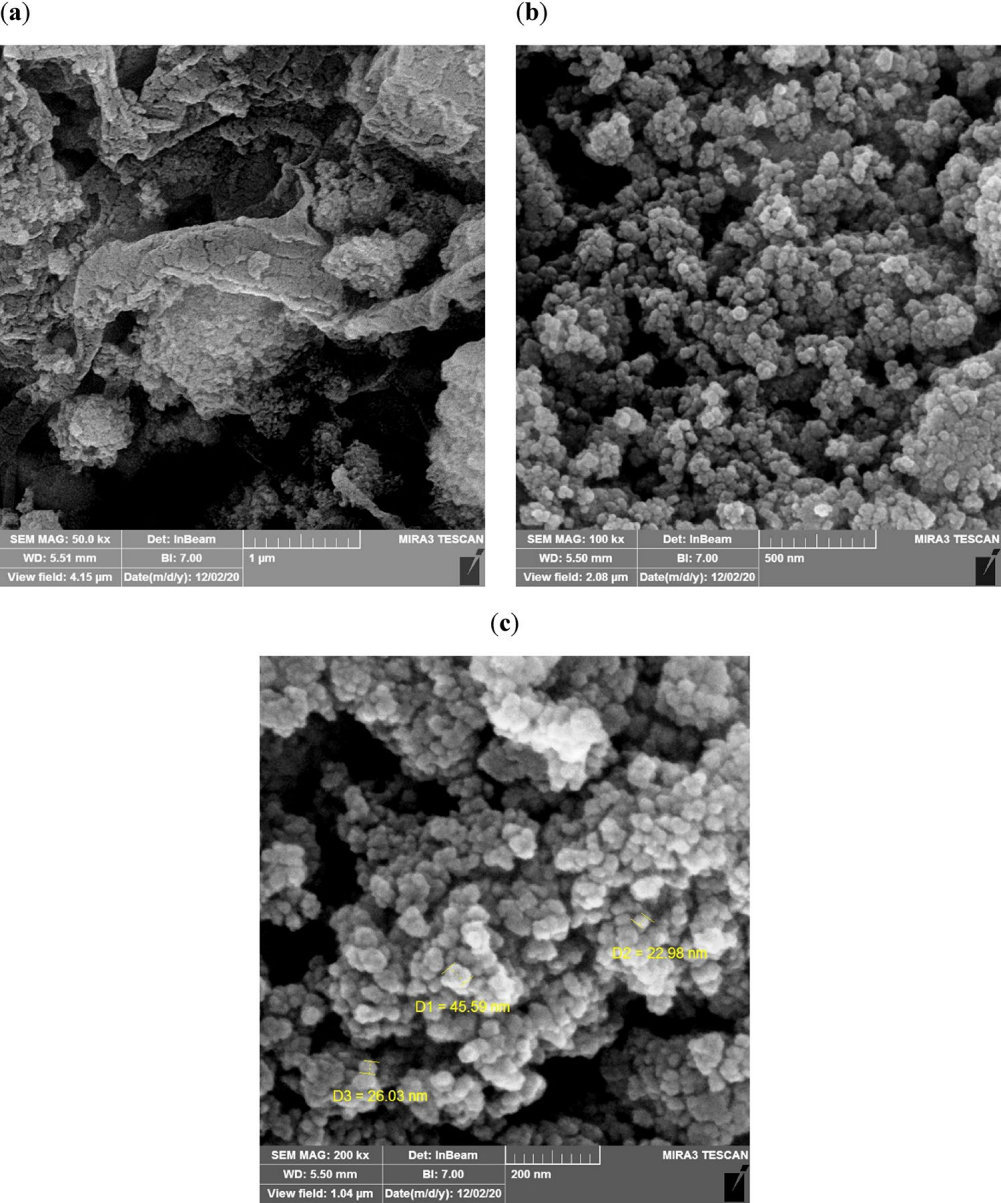
FESEM images of Fe_3_O_4_@SiO_2_@PTS-THEIC-(CH_2_)_3_OB(OH)_2_ magnetically recoverable catalyst (**1**).

To determine the size and morphology of the dendritic Fe_3_O_4_@SiO_2_@PTS-THEIC-(CH_2_)_3_OB(OH)_2_ catalyst (**1**), field emission scanning electron microscopy (FESEM) technique was used (Fig. 7). Interestingly, dendrons containing 3-propyl triethoxysilane (3-PTS), 1,3,5-tris(2-hydroxyethyl)isocyanurate and boric acid moieties are apparent (Fig. 7a–c). This may arise from the combination of both aromatic isocyanurate π-π stacking and boron-oxygen ligand interactions to afford supramolecular arrays of dendrons^1,19,128^. Furthermore, the obtained images shown in Fig. 7c illustrate that the structure of catalyst was made up of particles smaller than 46 nm.

### Investigation of the catalytic activity of dendritic Fe_3_O_4_@SiO_2_@PTS-THEIC-(CH_2_)_3_OB(OH)_2_ (1) for the synthesis of Hantzsch esters

**Figure 8:**
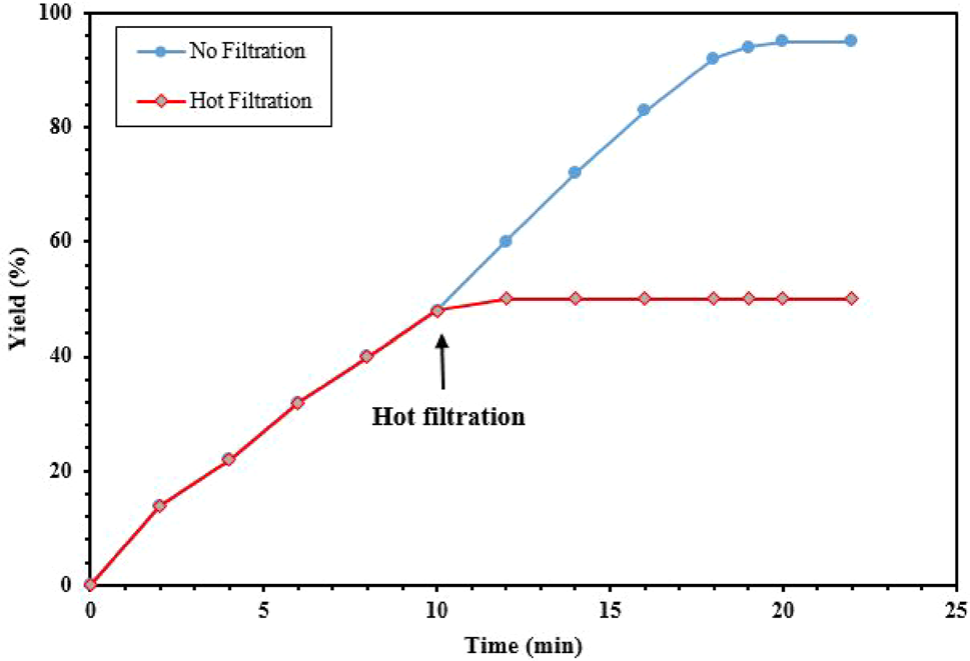
Hot filtration test for the synthesis of ethyl 4-(4-chlorophenyl)-2,7,7-trimethyl-5-oxo-1,4,5,6,7,8-hexahydroquinoline-3-carboxylate (**7a**) under optimized conditions (reproduced using the Microsoft Excel 2016).

After characterization of the dendritic Fe_3_O_4_@SiO_2_@PTS-THEIC-(CH_2_)_3_OB(OH)_2_ catalyst (**1**), the Hantzsch reaction for the synthesis of polyhydroacridine and polyhydroquinoline derivatives was chosen to examine the catalytic activity of Fe_3_O_4_@SiO_2_@PTS-THEIC-(CH_2_)_3_OB(OH)_2_ (**1**). For this purpose, the condensation of 4-chlorobenzaldehyde (**2a**, 1 mmol), dimedone (**3**), NH_4_OAc (**4**, 1 mmol) and/or ethyl acetoacetate (**6**, 1 mmol) were selected as the model reactions, for the synthesis of polyhydroacridine **5a** and polyhydroquinoline **7a**, respectively. The reactions were optimized considering different parameters such as the amount of catalyst loading, solvents and temperature. The results are reported in Table 1. Indeed, the reaction yield for the desired products 9-(4-chlorophenyl)-3,3,6,6-tetramethyl-3,4,6,7,9,10-hexahydroacridine-1,8(2*H*,5*H*)-dione (**5a**) or ethyl 4-(4-chlorophenyl)-2,7,7-trimethyl-5-oxo-1,4,5,6,7,8-hexahydroquinoline-3-carboxylate (**7a**) were trace in the absence of any catalyst in EtOH at room temperature (entry 1). However, low yields of the desired products **5a** and **7a** were obtained under reflux conditions (entry 2) after long times. Interestingly, the yields were improved significantly in the presence of dendritic Fe_3_O_4_@SiO_2_@PTS-THEIC-(CH_2_)_3_OB(OH)_2_ catalyst (**1**, entries 3–5). Further optimization of the reaction conditions illustrated that EtOH is the best solvent to promote the reaction with high efficiency for the synthesis of the desired products **5a** or **7a** (entries 6–12). The results of optimizing of the model reactions demonstrated that the optimal conditions for the reaction are 10 mg catalyst **1** loading in EtOH under reflux conditions. On the other hand, both boric acid and Fe_3_O_4_@SiO_2_@PTS-THEIC, as the components of the catalyst **1**, afforded moderate yields of the desired products **5a** and **7a** at same catalyst loading under optimized conditions (entries 13 and 14). Finally, hot filtration test (the Sheldon test) was performed to prove the heterogeneous nature of the catalyst **1**. During this test, the solid catalyst **1** was removed from the mixture of model reaction for producing **7a** by filtration after 10 min using an external magnet. Then, the obtained mixture was heated again for 10 min. The result showed that after removal of the magnetic catalyst **1**, the model reaction did not proceed significantly. Indeed, only 48% of the desired product **7a** was isolated after 1 h (Fig. 8).

**Table 1:** Optimization of the reaction of 4-chlorobenzaldeyde (**2a**), dimedone (**3**), NH_4_OAc (**4**) and/or ethyl acetoacetate (**6**) under different conditions (The chemical structures were drawn using ChemDraw Ultra 12.0 software developed by PerkinElmer)^a^.
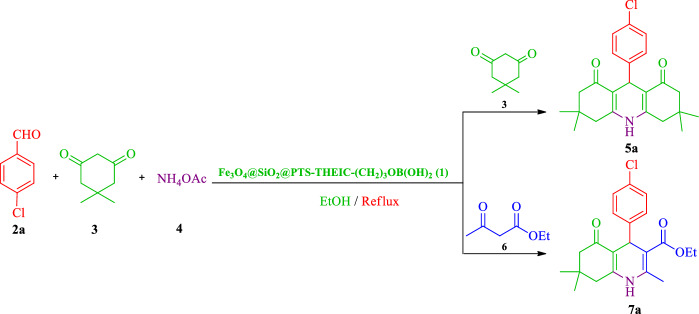

Entry	Catalyst **1** loading (mg)	Solvent	Temp. (°C)	Time (min)	Yield^b^ (%) Product **5a**	Time (min)	Yield (%) Product **7a**
1	–	EtOH	r.t	190	Trace	120	Trace
2	–	EtOH	Reflux	140	22	100	25
3	5	EtOH	Reflux	100	86	45	85
4	**10**	**EtOH**	**Reflux**	**60**	**92**	**20**	**95**
5	15	EtOH	Reflux	60	92	20	95
6	10	H_2_O	Reflux	110	67	70	64
7	10	CH_3_CN	Reflux	115	78	80	85
8	10	EtOH	r.t	100	76	90	80
9	10	H_2_O	r.t	130	70	100	64
10	10	EtOH	60 °C	90	84	60	84
11	10	H_2_O	60 °C	120	70	90	64
12	10	Solvent-Free	60 °C	100	82	60	86
13	10 (H_3_BO_3_)	EtOH	Reflux	60	61	20	66
14	10 (Fe_3_O_4_@SiO_2_@PTS-THEIC)	EtOH	Reflux	60	75	20	78

^a^Reaction conditions: 4-chlorobenzaldehyde (**2a**, 1 mmol), dimedone (**3**, 2 or 1 mmol), NH_4_OAc (**4**, 1.5 mmol) or ethyl acetoacetate (**6**, 1 mmol) in EtOH (2 ml); ^b^isolated yields.

**Scheme 2:**
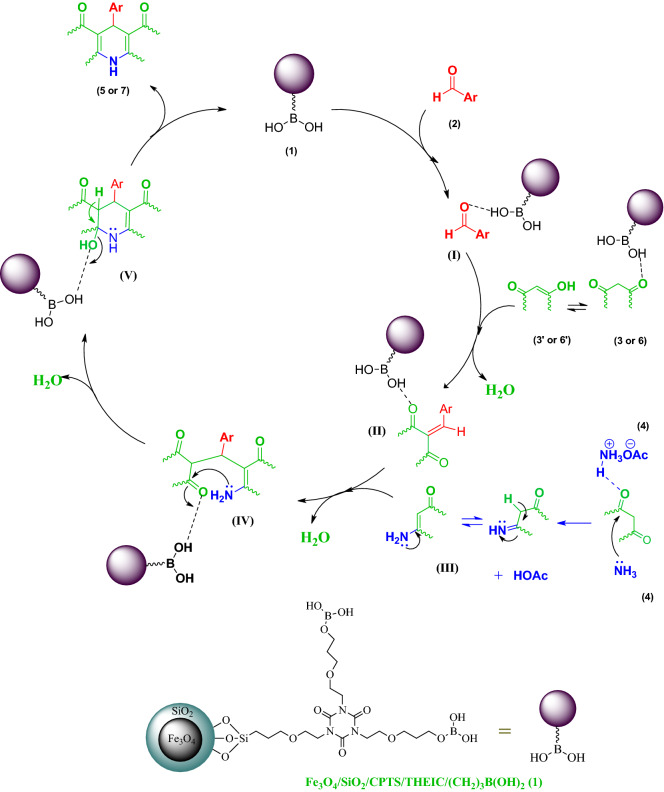
Plausible mechanism for the one-pot synthesis of polyhydroacridines **5** and polyhydroquinolines **7** catalyzed by the agnetically recoverable Fe_3_O_4_@SiO_2_@PTS-THEIC-(CH_2_)_3_OB(OH)_2_ catalyst (**1**, Drawn using the ChemDraw Ultra 12.0 software developed by PerkinElmer).

After finding the optimal conditions, the catalytic activity of Fe_3_O_4_@SiO_2_@PTS-THEIC-(CH_2_)_3_OB(OH)_2_ nanocatalyst (**1**) was further expanded to several other aromatic or heterocyclic aldehydes for the synthesis of other derivatives of PHAs **5a–o** and PHQs **7a–u**. As it is shown in Tables 2 and 3, the isolated yields of the desired products **6** or **8** were good to excellent in all studied cases under the optimized condition of reaction. In most cases, the products were obtained in similar periods of time and yields compared to the model reaction. Indeed, aldehydes including aromatic carbocyclic or heterocyclic substrates well survived under optimized conditions without formation of any by-products. It is noteworthy that aldehydes bearing electron-withdrawing groups or six-membered heterocycles almost reacted faster than substrates having electron-donating groups or five-membered heterocycles. This trend of reactivity was observed in both symmetric and asymmetric Hantzsch reaction to afford PHAs **5a–o** or PHQs **7a–u** derivatives, respectively. Furthermore, the α,β-unsaturated cinnamaldehyde (**2q**) or aliphatic butyraldehyde (**2r**) reacted in longer reaction times and afforded lower yields. These may be due to resonance and electron-releasing of the double bond and alkyl groups, respectively. All of these findings, led us to purpose a plausible mechanism depicted in Scheme 2.

**Table 2:** Fe_3_O_4_@SiO_2_@PTS-THEIC-(CH_2_)_3_OB(OH)_2_-catalyzed one-pot synthesis of polyhydroacridines **5a–o** from different aldehydes (**2a–o**), dimedone (**3**) and NH_4_OAc (**4**) under the optimized conditions (The chemical structures were drawn using ChemDraw Ultra 12.0 software developed by PerkinElmer)^a^.
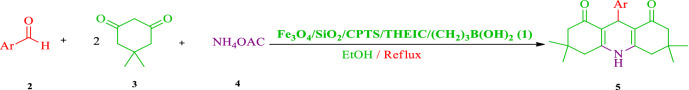

Entry	ArCHO **2**	Product **5**	Time (min)	Yield^b^ %	Mp (°C) Obs [Lit.]^c^
1		**5a**	60	92	311–313 [315–317]^129^
2		**5b**	90	80	197–200 [201–203]^130^
3		**5c**	75	80	321–323 [321]^131^
4		**5d**	45	93	311–314 [311–313]^131^
5		**5e**	60	80	308–310 [310–312]^132^
6		**5f.**	60	85	249–252 [249–251]^133^
7		**5g**	160	72	220–223 [223–225]^134^
8		**5h**	90	87	268–270 [273–275]^130^
9		**5i**	60	82	284–286 [284–286]^135^
10		**5j**	95	85	280–282 [282–283]^130^
11		**5k**	100	85	244–246 [246–248]^136^
12		**5l**	60	88	301–303 [298–300]^136^
13		**5m**	60	86	328–330 [320–325]^137^
14		**5n**	160	80	277–279 [278–279]^138^
15		**5o**	90	82	272–274 [274–276]^133^

^a^Reaction conditions: aldehyde (**2**, 1 mmol), dimedone (**3**, 2 mmol) and NH_4_OAc (**4**, 1.5 mmol) in EtOH (2 ml); ^b^isolated yields. ^c^All products are known and their structures were established from their spectral data and melting points compared to authentic samples or literature values.

**Table 3:** Fe_3_O_4_@SiO_2_@PTS-THEIC-(CH_2_)_3_OB(OH)_2_-catalyzed one-pot synthesis of polyhydroquinolines **7a–u** from different aldehydes (**2a–u**), dimedone (**3**), NH_4_OAc (**4**) and ethyl acetoacetate (**5**) under the optimized conditions (The chemical structures were drawn using ChemDraw Ultra 12.0 software developed by PerkinElmer)^a^.
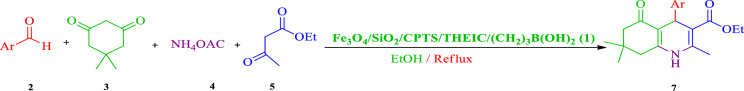

Entry	ArCHO **2**	Product **7**	Time (min)	Yield^b^ %	Mp (°C) Obs [Lit.]^c^
1		**7a**	20	95	243–245 [242–244]^115^
2		**7b**	20	92	249–251 [248–250]^139^
3		**7c**	45	89	234–236 [238–240]^139^
4		**7d**	45	92	196–198 [200–202]^140^
5		**7e**	45	84	182–184 [182–184]^115^
6		**7f.**	60	80	224–226 [226–228]^140^
7		**7g**	45	85	234–237 [238–241]^141^
8		**7h**	45	96	223–225 [224–226]^115^
9		**7i**	55	96	235–237 [239–242]^140^
10		**7j**	25	93	260–263 [263–265]^115^
11		**7k**	220	67	209–212 [208–211]^142^
12		**7l**	20	95	256–259 [255–257]^115^
13		**7m**	190	62	223–225 [225–227]^143^
14		**7n**	80	70	230–232 [233–235]^144^
15		**7o**	90	65	186–188 [184–186]^144^
16		**7p**	120	74	218–221 [223–225]^144^
17		**7q**	90	56	203–20 [204–205]^141^
18		**7r**	90	67	166–168 [165–167]^145^
19		**7t**	55	94	273–275 [274–276]^146^
20		**7u**	45	80	157–160 [157–160]^147^

^a^Reaction conditions: aldehyde (**2**, 1 mmol), dimedone (**3**, 1 mmol), NH_4_OAc (**4**, 1.5 mmol) and ethyl acetoacetate (**5**, 1 mmol) in EtOH (2 ml); ^b^isolated yields. ^c^All products are known and their structures were established from their spectral data and melting points compared to authentic samples or literature values.

**Figure 9:**
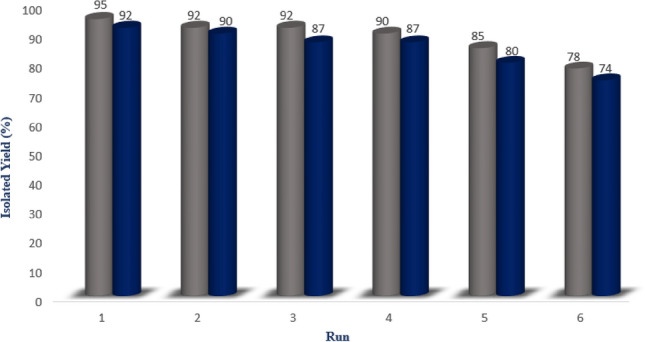
Recyclability of the dendritic Fe_3_O_4_@SiO_2_@CPTS-THEIC-(CH_2_)_3_OB(OH)_2_ nanocatalyst (**1**) for the synthesis of **5a** and **7a** (Drawn using the Microsoft Excel 2016).

**Figure 10:**
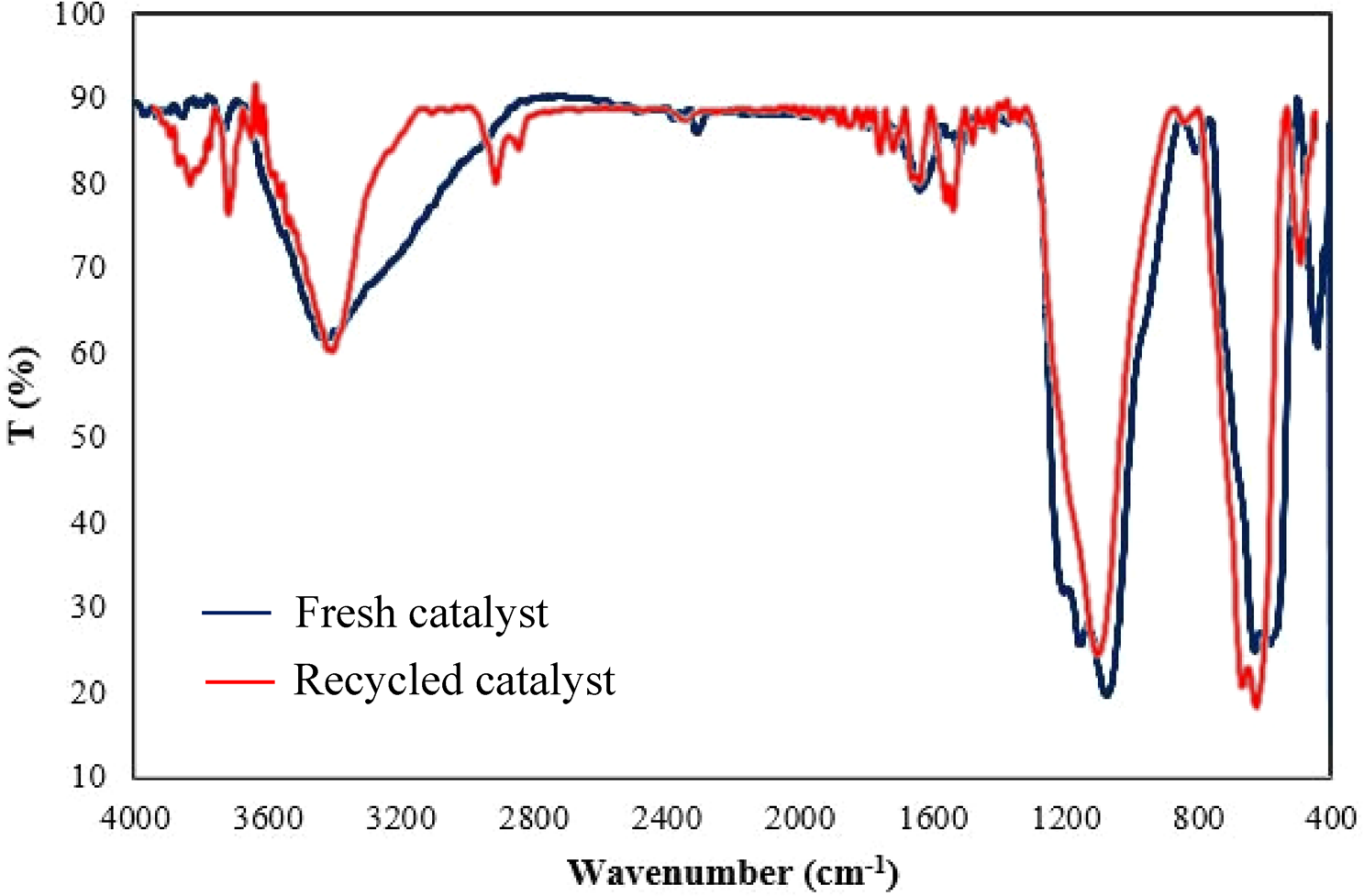
FTIR spectra of the fresh Fe_3_O_4_@SiO_2_@CPTS-THEIC-(CH_2_)_3_OB(OH)_2_ nanocatalyst (**1**) and the recycled sample after six consecutive runs for the synthesis of **5a** (reproduced using the Microsoft Excel 2016).

An important distinguishing feature of this magnetic dendritic nanocatalyst (**1**) beside easy separation from the reaction mixture is its recyclability. After the reaction was completed, the catalyst was separated and washed by acetone and hexane, respectively. Then, it was dried and reused in the model reactions for the next runs. The obtained results have been summarized in Fig. 9. These results show that this catalyst can be recovered and reused at least for five times in further runs under optimized conditions without a notable loss of its activity. Furthermore, comparison of the FTIR spectra of both fresh dendritic Fe_3_O_4_@SiO_2_@CPTS-THEIC-(CH_2_)_3_OB(OH)_2_ nanocatalyst (**1**) and the recycled sample after six consecutive runs for the synthesis of **5a** demonstrated that their structures are almost similar (Fig. 10).

Table 4 contains some of the formerly reported methods and representing their catalytic activity for the synthesis of polyhydroacridines and polyhydroquinolines to compare them with the dendritic Fe_3_O_4_@SiO_2_@CPTS-THEIC-(CH_2_)_3_OB(OH)_2_. These data clearly demonstrate that the nanocatalyst **1** is more active than other previously reported catalytic systems in terms of catalyst loading, product yield, required reaction time and avoiding the toxic solvents.

**Table 4:** Comparison of the synthesis of compounds **5a** and **7a** using the reported methods versus the present method.

Entry	Catalyst	Product	Catalyst Loading (mg)	Solvent	T °C	Time (min.)	Yield	Ref
1	KH_2_PO_4_	**5a**	5 mol%	EtOH/H_2_O	120	5 h	94	^146^
2	DABCO–PEG-400 ionic liquid	**5a**	80	–	115	12–14 h	92	^148^
3	Silica bonded *N*-propyl sulfamic acid	**5a**	30	EtOH	Reflux	2 h	86	^149^
4	Sawdust sulphonic acid	**5a**	50	EtOH	Reflux	1 h	90	^100^
5	Fe_3_O_4_@SiO_2_@PTS-THEIC-(CH_2_)_3_OB(OH)_2_	**5a**	10	EtOH	Reflux	1 h	92	This Work
6	L-proline	**7a**	10	EtOH	Reflux	360	92	^150^
7	Yb(OTf)_3_	**7a**	60	EtOH	25	300	90	^151^
8	PdRuNi@GO	**7a**	6	DMF	70	45	92	^24^
9	*p*‐Toluenesulfonic acid	**7a**	18	–	r.t	120	90	^152^
10	Fe_3_O_4_@B-MCM-41	**7a**	50	EtOH	Reflux	40	92	^118^
11	Silica Sulfuric Acid (SSA)	**7a**	80	–	60	45	93	^153^
12	PMO-ICS-PrSO_3_H	**7a**	20	EtOH	Reflux	20	95	^117^
13	Fe_3_O_4_@SiO_2_@PTS-THEIC-(CH_2_)_3_OB(OH)_2_	**7a**	10	EtOH	Reflux	20	95	This Work

### Experimental section

#### General information

All chemicals and reagents were provided by Merck or Aldrich chemical companies and used as received without any further purification, except for benzaldehyde which was used as a fresh distilled sample. FTIR spectra were recorded using KBr pellets on a Shimadzu FT IR-8400S spectrometer. Energy dispersive spectroscopy (EDS) was recorded on a SAMx instrument. The X-ray powder diffraction (XRD) data were collected on an X'Pert MPD Philips diffractometer with Cu radiation source (λ = 1.54050 Å) at 40 kV voltage and 40 mA current. Field emission scanning electron microscopy (FESEM) images were obtained using a MIRA3 instrument of TESCAN Company, Czech Republic. Thermal gravimetric analysis (TGA) and differential thermal analysis (DTA) were performed by means of a Bahr company STA 504 instrument. The BET specific surface area of the catalyst **1** was obtained using an equipment ASAP 2020 Micromeritics. Magnetic susceptibility measurements were taken out by using a Lakeshore VSM, 7410 series. Melting points were determined using an Electrothermal 9100 apparatus and are uncorrected. ^1^H NMR (500 MHz) spectra were obtained using a Bruker DRX-500 AVANCE spectrometer in CDCl_3_ at ambient temperature. Analytical TLC was carried out using Merck 0.2 mm silica gel 60 F-254 Al-plates and n-hexane: EtOAc, (3:1, v/v %) as eluent. All products are known and their structures were established by comparing the physical constants as well as FTIR and NMR spectroscopic data with authentic samples^120,122,141^.

#### ***Preparation of Fe***_***3***_***O***_***4***_***@SiO***_***2***_*** nanoparticles modified by (3-chloropropyl) trimethoxysilane (Fe***_***3***_***O***_***4***_***@SiO***_***2***_***@CPTS)***

The Fe_3_O_4_@SiO_2_@CPTS materials were prepared according to the reported methods in literature with a slight modification^56^.

#### ***Preparation of the dendritic Fe***_***3***_***O***_***4***_***@SiO***_***2***_***@CPTS@THEIC nanomaterials***

Fe_3_O_4_@SiO_2_@CPTS (1 g) was dispersed in toluene (30 ml) and KI (1.66 g) was added to the obtained mixture with the mechanical stirring at 80 °C for 1 h. Then, K_2_CO_3_ (1.38 g) and tris-(2-hydroxyethyl)-1,3,5-triazinane-2,4,6-trione (1 g) were added to the mixture and it was heated under reflux conditions for 8 h. The obtained solid was filtered off and washed with EtOH (5 ml) and then dried in an oven for 2 h.

#### ***Preparation of the dendritic Fe***_***3***_***O***_***4***_***@SiO***_***2***_***@PTS-THEIC-(CH***_***2***_***)***_***3***_***OB(OH)***_***2***_*** nanocatalyst (1)***

**Scheme 3:**
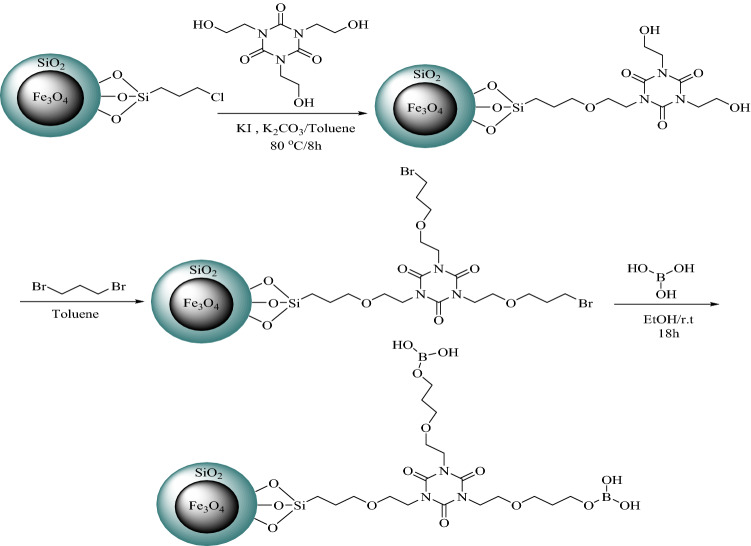
Schematic preparation of the dendritic Fe_3_O_4_@SiO_2_@PTS-THEIC-(CH_2_)_3_OB(OH)_2_ catalyst (**1**, Drawn using the ChemDraw Ultra 12.0 software developed by PerkinElmer**)**.

A mixture of Fe_3_O_4_@SiO_2_@CPTS@THEIC (1 g) and 1,3-dibromopropane (*d* = 1.98 g.cm^−3^, 2 ml) was added to toluene (15 ml) and heated at 40 °C for 12 h. The obtained solid was filtered off, washed with toluene (5 ml) and then dried in a vacuum oven at 60 °C for 2 h. The as-prepared solid and H_3_BO_3_ (1 g) were mixed in EtOH (30 ml) and the obtained mixture was stirred at room temperature for 18 h. After completion of the process, the obtained brown solid was filtered off and washed with EtOH (5 ml) on a Buchner funnel and then kept in a vacuum oven at 60 °C for 12 h. The complete procedure for the preparation of catalyst **1** has been represented in Scheme 3.

#### ***General procedure for the synthesis of 1,8-dioxoacridindione derivatives 5a–o catalyzed by magnetic dendritic Fe***_***3***_***O***_***4***_***@SiO***_***2***_***@PTS-THEIC-(CH***_***2***_***)***_***3***_***OB(OH)***_***2***_*** catalyst (1)***

In a 5 mL round-bottomed flask, a mixture of aldehyde (**2a–o**, 1 mmol), dimedone (**3**, 2 mmol, 0.28 g), NH_4_OAc (**4**, 1.5 mmol, 0.11 g) and Fe_3_O_4_@SiO_2_@PTS-THEIC-(CH_2_)_3_OB(OH)_2_ (1, 0.01 g) were added to EtOH 96% (2 mL). The obtained mixture was stirred under reflux conditions for the times indicated in Table 2. The progress of the reactions was monitored by TLC experiment (eluent; n-hexane: EtOAc, 3:1, v/v %). After completion of the reaction, EtOH (3 mL) was added to the mixture and it was heated to dissolve all organic compounds. Then, the catalyst **1** was easily separated by an external magnet and the solution was filtered. The filtrate was kept at room temperature and the crystals were collected by filtration to afford 1,8-dioxoacridindione derivatives **5a–o** in high purity.

#### ***General procedure for the synthesis of polyhydroquinoline derivatives 7a–u catalyzed by magnetic dendritic Fe***_***3***_***O***_***4***_***@SiO***_***2***_***@PTS-THEIC-(CH***_***2***_***)***_***3***_***OB(OH)***_***2***_*** catalyst (1)***

In a 5 mL round-bottomed flask, a mixture of aldehyde (**2a–u**, 1 mmol), dimedone (**3**, 1 mmol, 0.14 g), NH_4_OAc (**4**, 1.5 mmol, 0.11 g), ethyl acetoacetate (**5**, 1 mmol, 0.13 g) and Fe_3_O_4_@SiO_2_@PTS-THEIC-(CH_2_)_3_OB(OH)_2_ (**1**, 0.01 g) were added to EtOH 96% (2 ml). The obtained mixture was stirred under reflux conditions for times indicated in Table 3. The progress of the reactions was monitored by TLC experiment (eluent; n-hexane: EtOAc, 3:1, v/v %). After completion of the reaction, EtOH (3 mL) was added to the mixture and it was heated to dissolve all organic compounds. Then, the catalyst **1** was easily separated by an external magnet and the solution was filtered. The filtrate was kept at room temperature and the crystals were collected by filtration to afford polyhydroquinoline derivatives **7a–u** in high purity.

### Selected spectral data

#### 9-(4-Chlorophenyl)-3,3,6,6-tetramethyl-3,4,6,7,9,10-hexahydro-1,8(2*H*,5*H*)-acridinedione (5a)

Pale yellow solid; m.p. = 310–312 °C; FT-IR (KBr, cm^−1^): 3282, 3176, 3060, 2954, 2875,1650, 1608, 1492, 1365, 1220, 1147, 1089, 1014, 840, 761, 597, 526; ^1^H NMR (500 MHz, CDCl_3_): δ (ppm): 0.98 (s, 6H, 2CH_3_), 1.10 (s, 6H, 2CH_3_), 2.19–2.37 (8H, m, 4CH_2_), 5.06 (s, 1H, CH), 7.17 (d, 2H, Ar–H), 7.28 (d, 2H, Ar–H), 6.97 (s, 1H, NH).

#### 3,3,6,6-Tetramethyl-9-(pyridin-2-yl)-3,4,6,7,9,10-hexahydroacridine-1,8(2*H*,5*H*)-dione (5i):

Pale yellow solid; m.p. = 284–286 °C; FT-IR (KBr, cm^−1^): 3604, 3519, 3440, 3284, 2875, 1637, 1600, 1477, 1365, 1218, 1139, 995, 744, 563; ^1^H NMR (500 MHz, CDCl_3_): δ (ppm): 0.98 (s, 6H, 2CH_3_), 1.07 (s, 6H, 2CH_3_), 2.12–2.46 (8H, m, 4CH_2_), 5.22 (s, 1H, CH), 7.51–7.58 (t, 3H, Ar–H), 8.41 (d, 1H, Ar–H), 6.97 (s, 1H, NH).

#### Ethyl 4-(4-methoxyphenyl)-2,7,7-trimethyl-5-oxo-1,4,5,6,7,8-hexahydroquinoline-3-carboxylate (7l)

Pale yellow solid; mp.: 255–260 °C; FT-IR (KBr, cm^−1^): 3278, 3203, 3076, 2956, 1699, 1604, 1496, 1379, 1276, 1218, 1070, 1031, 842, 765, 536; ^1^H NMR (500 MHz, CDCl_3_): δ (ppm): 0.92 (s, 3H, CH_3_), 1.04 (s, 3H, CH_3_), 1.20 (t, 3H, *J* = 7.2 Hz, CH_3 (OEt)_), 2.11–2.28 (m, 4H, CH_2_), 2.33 (s, 3H, CH_3_), 3.71 (s, 3H, OCH_3_), 4.03–4.07 (q, 2H, *J* = 7.2 Hz, CH_2 (OEt)_), 4.98 (s, 1H, CH _benzylic_), 6.43 (br s, 1H, NH), 6.71–6.73 (d, 2H, *J* = 8.2 Hz, Ar–H), 7.21 (d, 2H, *J* = 8.2 Hz, Ar–H).

#### Ethyl 2,7,7-trimethyl-4-(3-nitrophenyl)-5-oxo-1,4,5,6,7,8-hexahydroquinoline-3-carboxylate (7e)

Pale yellow solid; m.p. = 180–184 °C; FT-IR (KBr, cm^−1^): 3276, 3193, 2964, 1703, 1604, 1490, 1379, 1278, 1215, 1143, 1070, 1022, 829, 754, 690, 507; ^1^H NMR (500 MHz, CDCl_3_): δ (ppm): 0.93 (s, 3H, CH_3_), 1.09 (s, 3H, CH_3_), 1.19 (t, 3H, *J* = 7.2 Hz, CH_3 (OEt)_), 2.13–2.40 ( 7H, s CH_3_, m 2CH_2_), 4.03–4.07 (q, 2H, *J* = 7.2 Hz, CH_2 (OEt)_), 5.15 (s, 1H, CH_benzylic_), 5.98 (s, 1H, NH), 7.35–8.10 (m, 2H, Ar–H) (Supplementary Information 1).

### Conclusions

In conclusion, the multifunctional dendritic nanocatalyst containing boric acid and 1,3,5-tris(2-hydroxyethyl)isocyanurate covalently attached to core–shell silica-coated magnetite (Fe_3_O_4_@SiO_2_@PTS-THEIC-(CH_2_)_3_OB(OH)_2_) was prepared and properly characterized for the first time. It was found that the combination of both aromatic π–π stacking and boron–oxygen ligand interactions affords supramolecular arrays of dendrons. The use of boric acid makes this dendritic catalyst a green choice from corrosion, recyclability and cost points of view. The magnetic dendritic catalyst was used, as a mild and recyclable catalyst, for the one-pot efficient synthesis of polyhydroacridines and polyhydroquinolines through MCR strategy in EtOH as a green solvent. Indeed, very low catalyst loading, short reaction times, mild reaction conditions, high to excellent yields, reusability of the catalyst, ease of separation by an external magnetic field, and the use of nontoxic materials for the preparation of the catalyst are among other advantages of this protocol. Further exploring of this magnetic dendritic magnetic catalyst for other organic transformations is underway in our research lab and would be presented in due course.

## Supplementary Information

Supplementary Information 1.
