# Supplementary material for: Dendrons containing boric acid and 1,3,5-tris(2-hydroxyethyl)isocyanurate covalently attached to silica-coated magnetite for the expeditious synthesis of Hantzsch esters
Source: Sci Rep. 2021 Jan 27;11:2399. doi: 10.1038/s41598-020-80884-z (PMC7840758; doi:10.1038/s41598-020-80884-z)
Supplement: Supplementary file 1 — Supplementary Information 1. [file 41598_2020_80884_MOESM1_ESM.docx]

***“Electronic Supplementary Information”***

***Scientific Reports - SpringerNature***

**Dendrons containing boric acid and 1,3,5-tris(2-hydroxyethyl)isocyanurate covalently attached to silica-coated magnetite for the expeditious synthesis of Hantzsch esters**

Mahsa Sam, Mohammad G. Dekamin*, Zahra Alirezvani

*E-mail:* [*mdekamin@iust.ac.ir*](mailto:mdekamin@iust.ac.ir)

Scheme 1. Schematic representation of the Fe_3_O_4_@SiO_2_@PTS-THEIC-(CH_2_)_3_OB(OH)_2_ catalyst (1) and its catalytic activity in the one-pot synthesis of polyhydroacridines (5) and polyhydroquinolines (7) through multicomponent reaction (MCR) strategy (Drawn using the ChemDraw Ultra 12.0 software developed by PerkinElmer).

**Scheme 2** Plausible mechanism for the one-pot synthesis of polyhydroacridines **5** and polyhydroquinolines **7** catalyzed by the agnetically recoverable Fe_3_O_4_@SiO_2_@PTS-THEIC-(CH_2_)_3_OB(OH)_2_ catalyst (**1**, Drawn using the ChemDraw Ultra 12.0 software developed by PerkinElmer**)**.

**.**

**Experimental Section**

***General Information***

All chemicals and reagents were provided by Merck or Aldrich chemical companies and used as received without any further purification, except for benzaldehyde which was used as a fresh distilled sample. FTIR spectra were recorded, as KBr pellets, on a Shimadzu FT IR-8400S spectrometer. Energy Dispersive Spectroscopy (EDS) was recorded on a SAMx instrument. The X-ray powder diffraction (XRD) data were collected on an X'Pert MPD Philips diffractometer with Cu radiation source (λ = 1.54050 Å) at 40 kV voltage and 40 mA current. Field emission scanning electron microscopy (FESEM) images were obtained using a MIRA3 instrument of TESCAN Company, Czech Republic. Thermal gravimetric analysis (TGA) and differential thermal analysis (DTA) were performed by means of a Bahr company STA 504 instrument. The BET specific surface area of the catalyst **1** was obtained using an equipment ASAP 2020 Micromeritics. Magnetic susceptibility measurements were taken out by using a Lakeshore VSM, 7410 series. Melting points were determined using an Electrothermal 9100 apparatus and are uncorrected. ^1^H NMR (500 MHz) spectra were obtained using a Bruker DRX-500 AVANCE spectrometer in CDCl_3_ at ambient temperature. Analytical TLC was carried out using Merck 0.2 mm silica gel 60 F-254 Al-plates and n-hexane: EtOAc, (3:1, v/v %) as eluent. All products are known and their structures were established by comparing the physical constants as well as FTIR and NMR spectroscopic data with authentic samples.

***Preparation of Fe_3_O_4_@SiO_2_ nanoparticles modified by (3-chloropropyl) trimethoxysilane (Fe_3_O_4_@SiO_2_@CPTS)***

The Fe_3_O_4_@SiO_2_@CPTS materials were prepared according to the reported methods in literature with a slight modification described in our previous paper (Reference: Ishani, M., Dekamin, M. G. & Alirezvani, Z., *Journal of Colloid Interface Science* **521**, 232-241 (2018)).

***Preparation of the dendritic Fe_3_O_4_@SiO_2_@CPTS@THEIC nanomaterials***

Fe_3_O_4_@SiO_2_@CPTS (1 g) was dispersed in toluene (30 ml) and KI (1.66 g) was added to the obtained mixture with the mechanical stirring at 80 °C for 1 h. Then, K_2_CO_3_ (1.38 g) and tris-(2-hydroxyethyl)-1,3,5-triazinane-2,4,6-trione (1 g) were added to the mixture and it was heated under reflux conditions for 8 h. The obtained solid was filtered off and washed with EtOH (5 ml) and then dried in an oven for 2 h.

*Preparation of the dendritic Fe_3_O_4_@SiO_2_@PTS-THEIC-(CH_2_)_3_OB(OH)_2_ nanocatalyst (1)*

A mixture of Fe_3_O_4_@SiO_2_@CPTS@THEIC (1 g) and 1,3-dibromopropane (*d* = 1.98 g.cm^-3^, 2 ml) was added to toluene (15 ml) and heated at 40 °C for 12 h. The obtained solid was filtered off, washed with toluene (5 ml) and then dried in a vacuum oven at 60 °C for 2 h. The as-prepared solid and H_3_BO_3_ (1 g) were mixed in EtOH (30 ml) and the obtained mixture was stirred at room temperature for 18 h. After completion of the process, the obtained brown solid was filtered off and washed with EtOH (5 ml) on a Buchner funnel and then kept in a vacuum oven at 60 °C for 12 h. The complete procedure for the preparation of catalyst **1** has been represented in **Scheme 3**.

**Scheme 3.** Schematic preparation of the dendritic Fe_3_O_4_@SiO_2_@PTS-THEIC-(CH_2_)_3_OB(OH)_2_ catalyst (**1**, Drawn using the ChemDraw Ultra 12.0 software developed by PerkinElmer**)**.

| 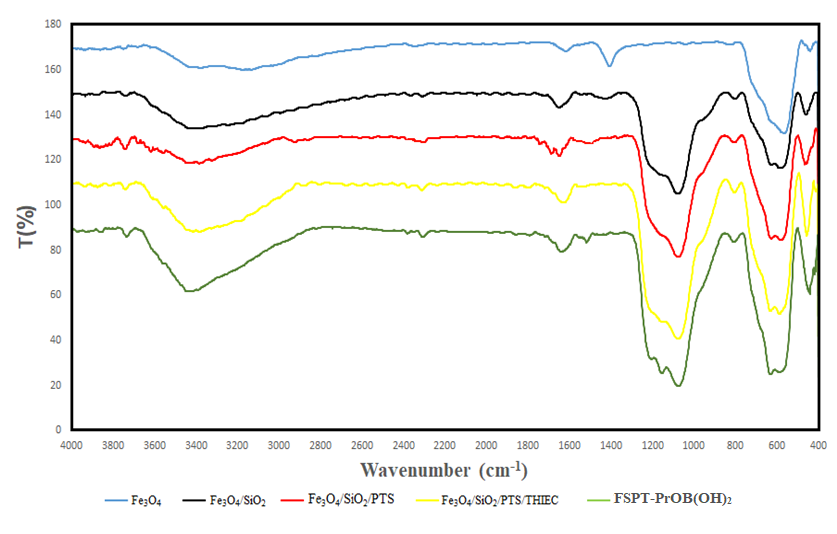 |
| --- |

**Fig. 10.** FTIR spectra of the fresh Fe_3_O_4_@SiO_2_@CPTS-THEIC-(CH_2_)_3_OB(OH)_2_ nanocatalyst (**1**) and the recycled sample after six consecutive runs for the synthesis of **5a** (reproduced using the Microsoft Excel 2016).

.

| 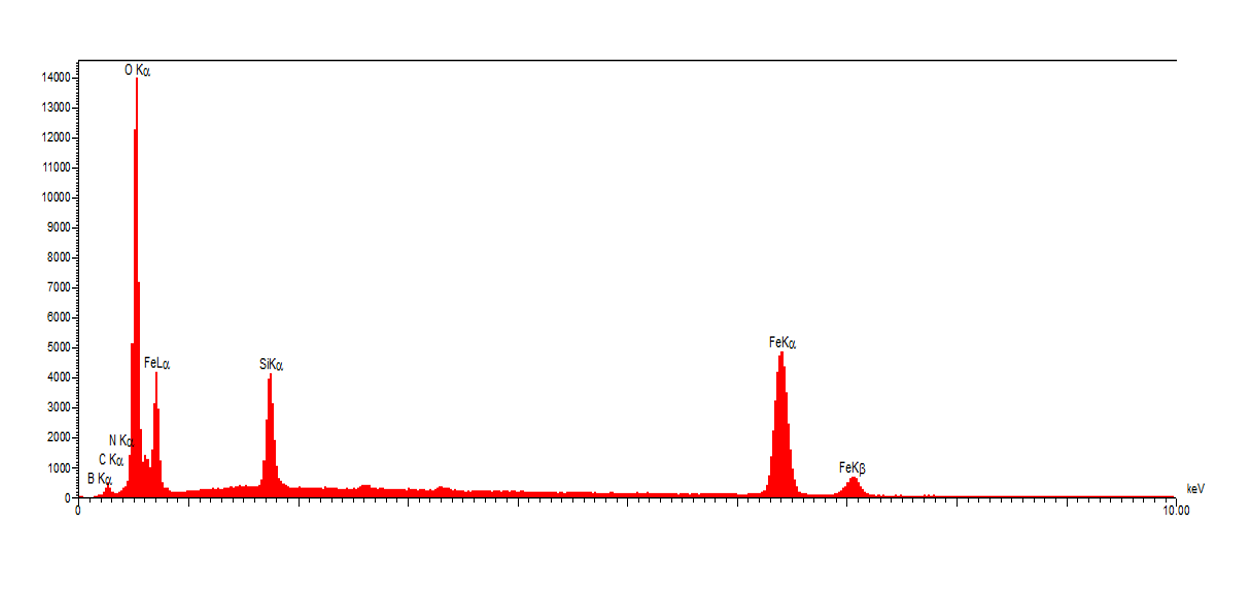 |
| --- |

**Fig. 2.** Energy dispersive spectroscopy (EDX) analysis of the magnetic Fe_3_O_4_@SiO_2_@PTS-THEIC-(CH_2_)_3_OB(OH)_2_ catalyst (**1**).


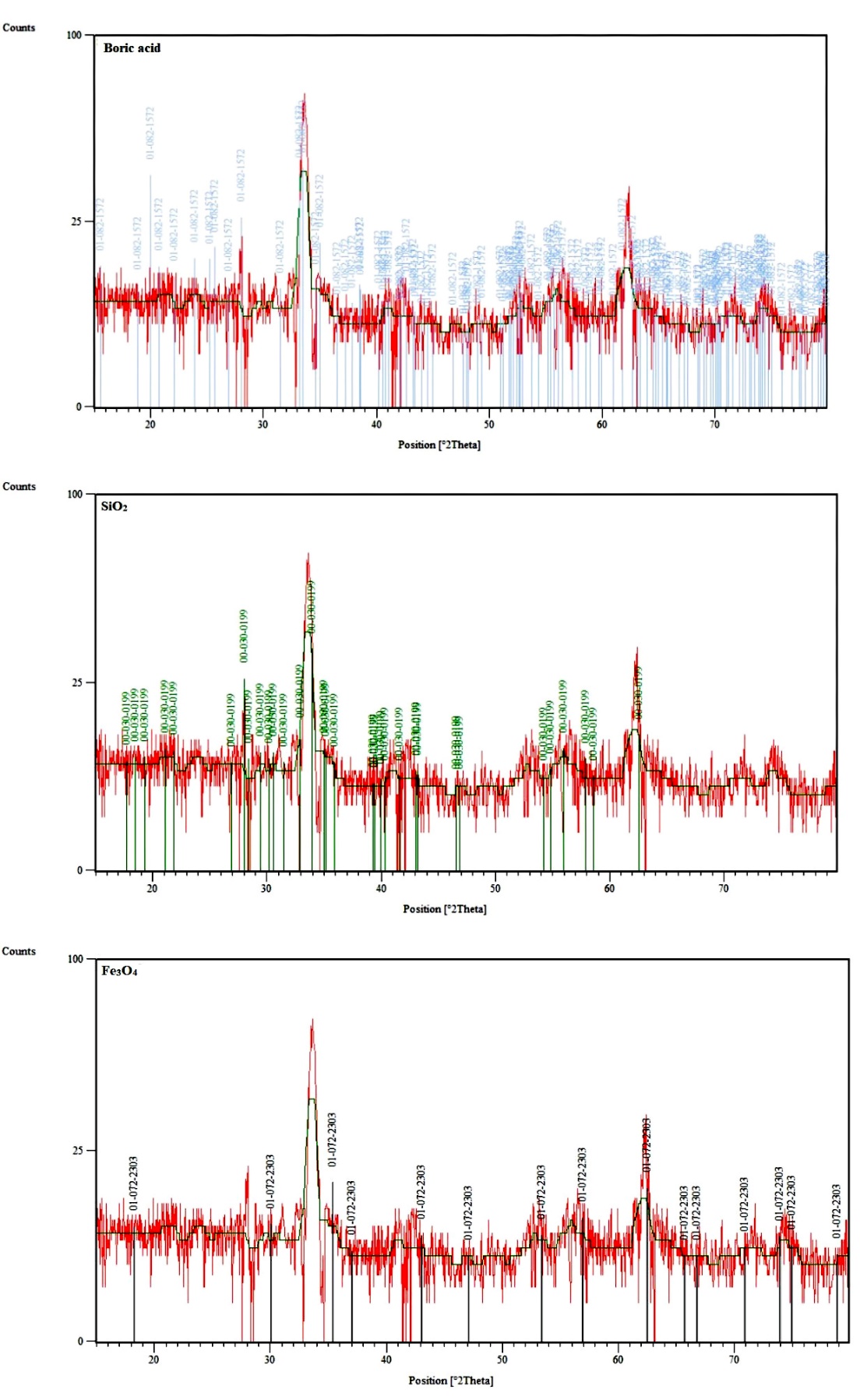


**Fig. 3.** X-Ray diffraction (XRD) pattern of the magnetic dendritic Fe_3_O_4_@SiO_2_@PTS-THEIC-(CH_2_)_3_OB(OH)_2_ catalyst (**1**, the individual reference card numbers of the catalyst **1** components were collected from the X'pert HighScore Plus version 2.1 software developed by the PANalytical B.V.).

| 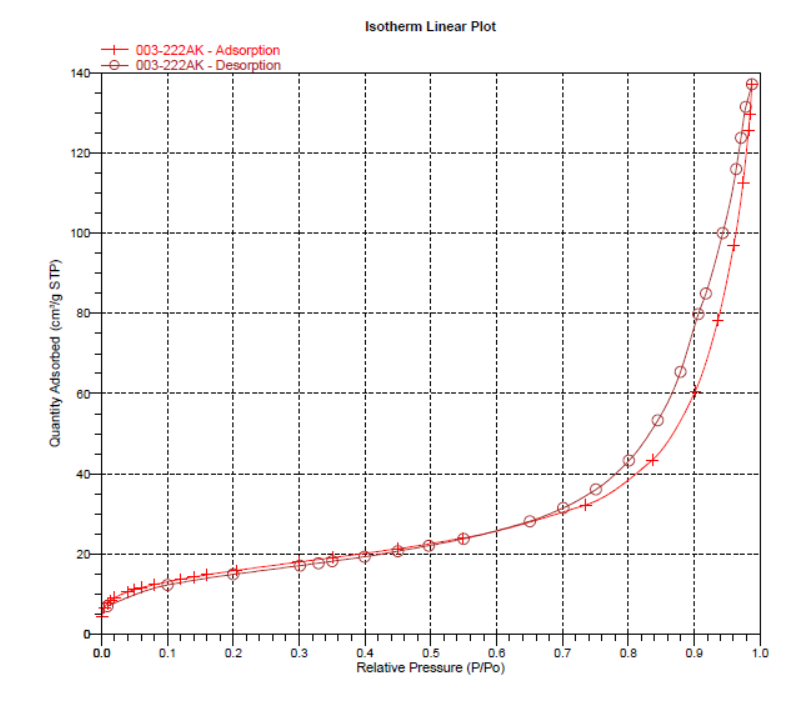 |
| --- |

**Fig. 4.** Nitrogen adsorption–desorption isotherm (BET) of the magnetic Fe_3_O_4_@SiO_2_@PTS-THEIC-(CH_2_)_3_OB(OH)_2_ catalyst (**1**).

**a)**

| **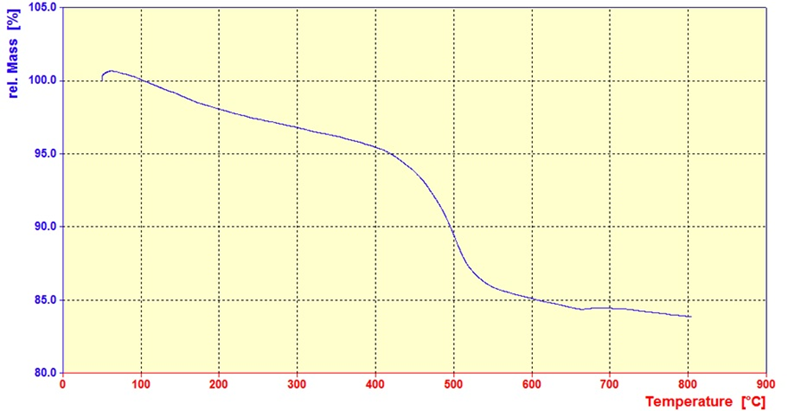**  **b)** |
| --- |
| **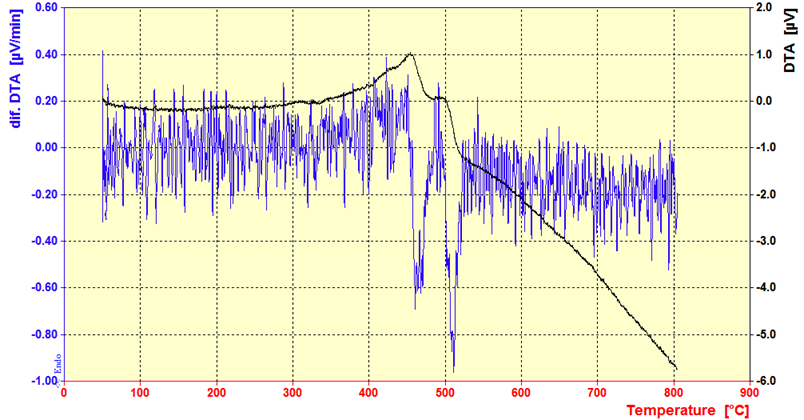** |

**Fig. 5.** a) Thermal gravimetric analysis (TGA) and b) differential thermal analysis (DTA) curves of the magnetic dendritic Fe_3_O_4_@SiO_2_@PTS-THEIC-(CH_2_)_3_OB(OH)_2_ catalyst (**1**).

| 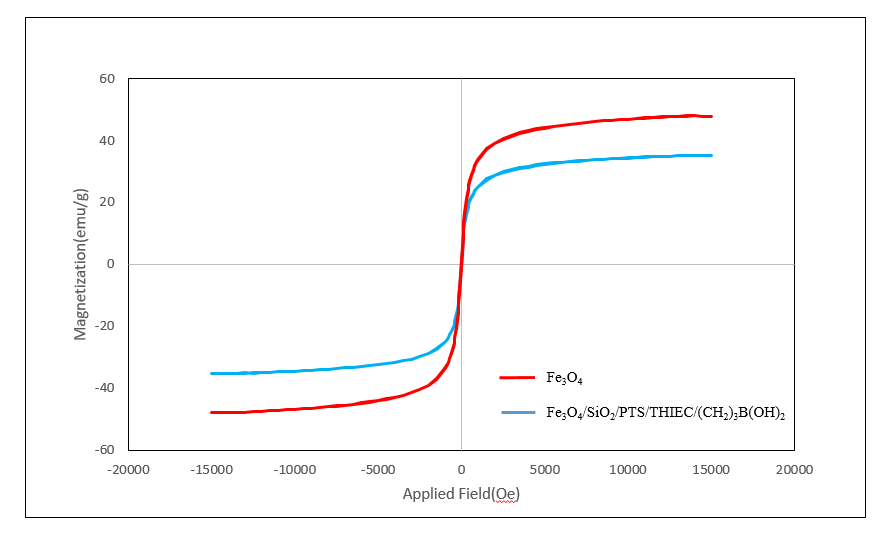 |
| --- |

**Fig. 6.** VSM analysis of the magnetic dendritic Fe_3_O_4_@SiO_2_@PTS-THEIC-(CH_2_)_3_OB(OH)_2_ catalyst (**1,** reproduced using the Microsoft Excel 2016).

| (**a**)  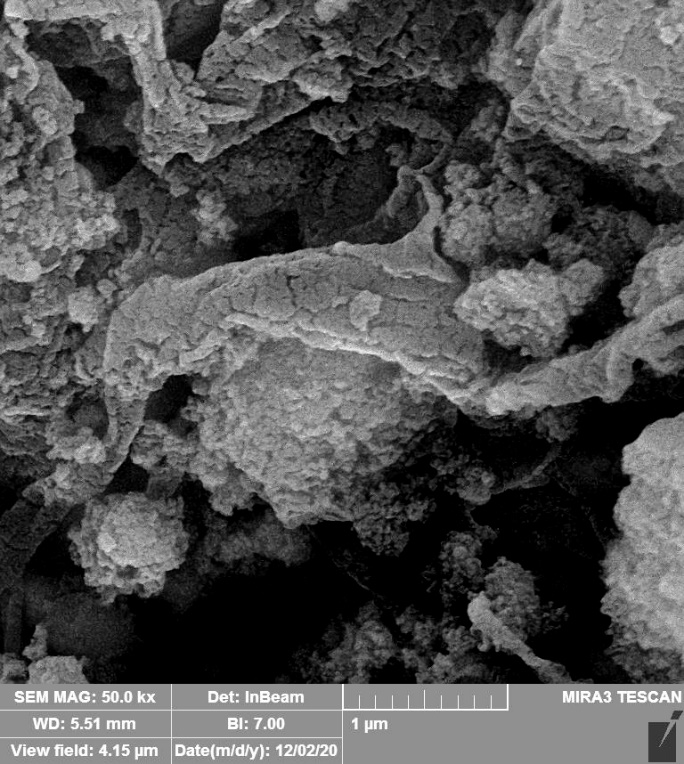 | (**b**)  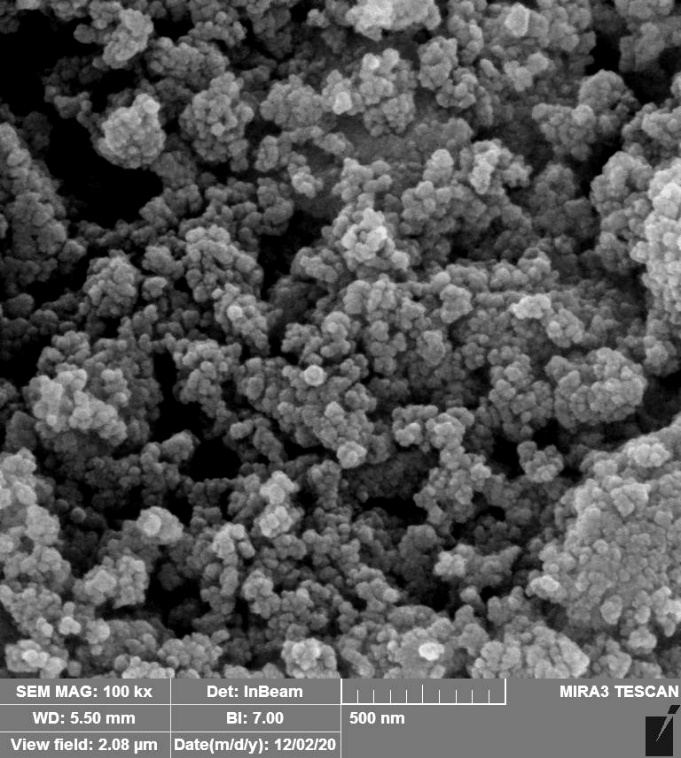 |
| --- | --- |
| (**c**)  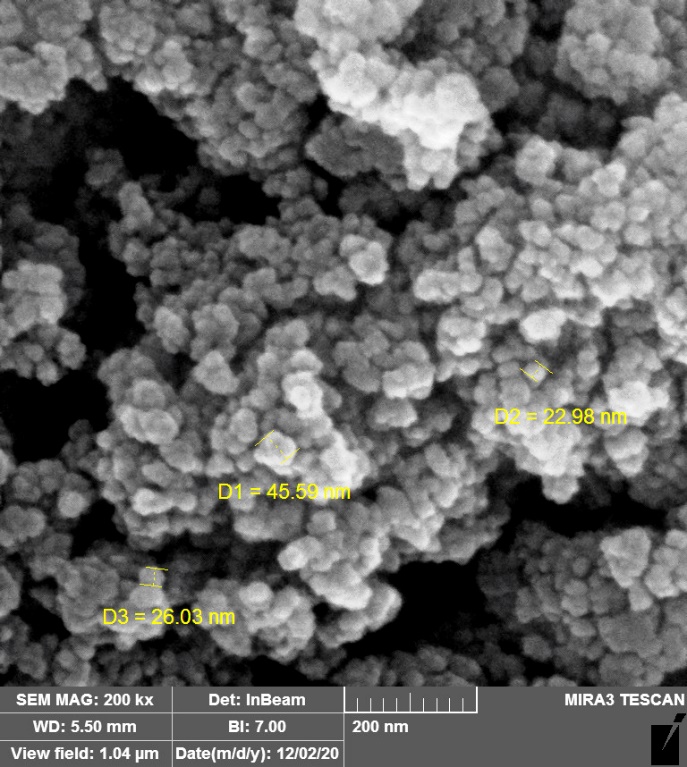 | |

**Fig. 7.** FESEM images of Fe_3_O_4_@SiO_2_@PTS-THEIC-(CH_2_)_3_OB(OH)_2_ magnetically recoverable catalyst (**1**).

| 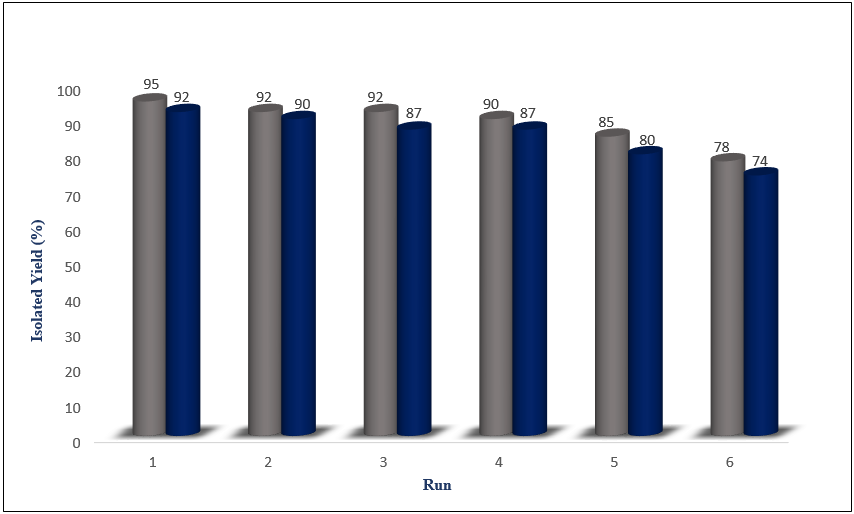 |
| --- |

**Fig. 8.** Recyclability of the dendritic Fe_3_O_4_@SiO_2_@CPTS-THEIC-(CH_2_)_3_OB(OH)_2_ nanocatalyst (**1**) for the synthesis of **5a** and **7a** (Drawn using the Microsoft Excel 2016).


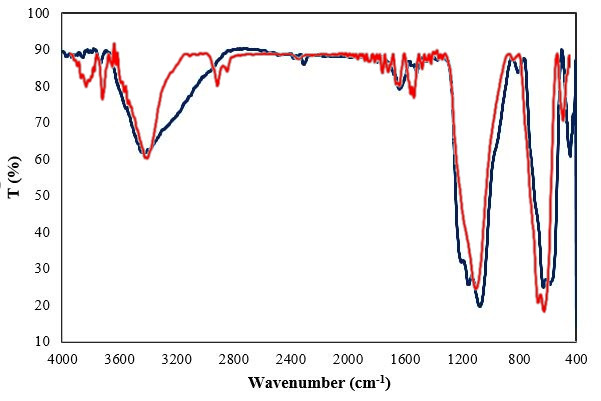


Fresh catalyst

Recycled catalyst

**Fig. 9.** FTIR spectra of the fresh Fe_3_O_4_@SiO_2_@CPTS-THEIC-(CH_2_)_3_OB(OH)_2_ nanocatalyst (**1**) and the recycled sample after six consecutive runs for the synthesis of **5a** (reproduced using the Microsoft Excel 2016).

**Table 1.** Optimization of the reaction of 4-chlorobenzaldeyde (**2a**), dimedone (**3**), NH_4_OAc (**4**) and ethyl acetoacetate (**5**) under different conditions (The chemical structures were drawn using ChemDraw Ultra 12.0 software developed by PerkinElmer).^a^

| Entry | Catalyst **1** loading (mg) | Solvent | Temp. (°C) | Time (min) | Yield^b^ (%)  Product **5a** | Time (min) | Yield (%)  Product **7a** |
| --- | --- | --- | --- | --- | --- | --- | --- |
| 1 | - | EtOH | r.t | 190 | Trace | 120 | Trace |
| 2 | - | EtOH | Reflux | 140 | 22 | 100 | 25 |
| 3 | 5 | EtOH | Reflux | 100 | 86 | 45 | 85 |
| 4 | **10** | **EtOH** | **Reflux** | **60** | **92** | **20** | **95** |
| 5 | 15 | EtOH | Reflux | 60 | 92 | 20 | 95 |
| 6 | 10 | H_2_O | Reflux | 110 | 67 | 70 | 64 |
| 7 | 10 | CH_3_CN | Reflux | 115 | 78 | 80 | 85 |
| 8 | 10 | EtOH | r.t | 100 | 76 | 90 | 80 |
| 9 | 10 | H_2_O | r.t | 130 | 70 | 100 | 64 |
| 10 | 10 | EtOH | 60 °C | 90 | 84 | 60 | 84 |
| 11 | 10 | H_2_O | 60 °C | 120 | 70 | 90 | 64 |
| 12 | 10 | Solvent-Free | 60 °C | 100 | 82 | 60 | 86 |
| ^a^Reaction conditions: 4-chlorobenzaldehyde (**2a**, 1 mmol), dimedone (**3**, 1 mmol), NH_4_OAc (**4**, 1.5 mmol) or ethyl acetoacetate (**5**, 1 mmol) in EtOH (2 ml); ^b^isolated yields. | | | | | | | |

**Table 2** Fe_3_O_4_@SiO_2_@PTS-THEIC-(CH_2_)_3_OB(OH)_2_-catalyzed one-pot synthesis of polyhydroacridines **5a-o** from different aldehydes (**2a-o**), dimedone (**3**) and NH_4_OAc (**4**) under the optimized conditions (The chemical structures were drawn using ChemDraw Ultra 12.0 software developed by PerkinElmer)^a^

| Entry | | ArCHO **2** | | Product **5** | | Time (min) | | Yield^b^ % | | Mp (°C)  Obs [Lit.]^c^ | |  |
| --- | --- | --- | --- | --- | --- | --- | --- | --- | --- | --- | --- | --- |
| 1 |   (**2a**) | | **5a** | | | 60 | | 92 | | 311-313 [315-317](Zarei and Akhlaghinia 2017) | |  |
| 2 |   (**2b**) | | **5b** | | | 90 | | 80 | | 197-200 [201-203](Fekri and Nikpassand 2016) | |  |
| 3 |   (**2c**) | | **5c** | | | 75 | | 80 | | 321-323 [321](Rostamizadeh, Amirahmadi et al. 2012) | |  |
| 4 |   (**2d**) | | **5d** | | | 45 | | 93 | | 311-314 [311-313](Rostamizadeh, Amirahmadi et al. 2012) | |  |
| 5 |   (**2e**) | | **5e** | | | 60 | | 80 | | 308-310 [310-312](BAKIBAEV, FILIMONOV et al. 1991) | |  |
| 6 |   (**2f**) | | | | **5f** | | 60 | | 85 | | 249-252 [249-251](Mahesh, Guruswamy et al. 2015) | |
| 7 |   (**2g**) | | **5g** | | | 160 | | 72 | | 220-223 [223-225](Ramesh, Pasha et al. 2014) | |  |
| 8 |   (**2h**) | | **5h** | | | 90 | | 87 | | 268-270 [273-275](Fekri and Nikpassand 2016) | |  |
| 9 |   (**2i**) | | **5i** | | | 60 | | 82 | | 284-286 [284-286](Das, Dutta et al. 2014) | |  |
| 10 |   (**2j**) | | **5j** | | | 95 | | 85 | | 280-282 [282-283](Fekri and Nikpassand 2016) | |  |
| 11 |   (**2k**) | | **5k** | | | 100 | | 85 | | 244-246 [246-248](Afsar, Zolfigol et al. 2018) | |  |
| 12 |   (**2l**) | | **5l** | | | 60 | | 88 | | 301-303 [298-300](Afsar, Zolfigol et al. 2018) | |  |
| 13 |   (**2m**) | | **5m** | | | 60 | | 86 | | 328-330 [320-325](Nasresfahani and Kassaee 2015) | |  |
| 14 |   (**2n**) | | **5n** | | | 160 | | 80 | | 277-279 [278-279](Suarez, Loupy et al. 1999) | |  |
| 15 |   (**2o**) | | **5o** | | | 90 | | 82 | | 272-274 [274-276](Mahesh, Guruswamy et al. 2015) | |  |
| ^a^Reaction conditions: aldehyde (**2**, 1 mmol), dimedone (**3**, 2 mmol) and NH_4_OAc (**4**, 1.5 mmol) in EtOH (2 ml); ^b^isolated yields. ^c^All products are known and their structures were established from their spectral data and melting points compared to authentic samples or literature values. | | | | | | | | | | | |  |

**Table 3** Fe_3_O_4_@SiO_2_@PTS-THEIC-(CH_2_)_3_OB(OH)_2_-catalyzed one-pot synthesis of polyhydroquinolines **7a-u** from different aldehydes (**2a-u**), dimedone (**3**), NH_4_OAc (**4**) and ethyl acetoacetate (**5**) under the optimized conditions (The chemical structures were drawn using ChemDraw Ultra 12.0 software developed by PerkinElmer)^a^

| Entry | ArCHO **2** | Product **7** | Time (min) | Yield^b^ % | Mp (°C)  Obs [Lit.]^c^ |
| --- | --- | --- | --- | --- | --- |
| 1 |   (**2a**) | **7a** | 20 | 95 | 243-245 [242-244](Zolfigol and Yarie 2015) |
| 2 |   (**2b**) | **7b** | 20 | 92 | 249-251 [248-250](Goli-Jolodar, Shirini et al. 2016) |
| 3 |   (**2c**) | **7c** | 45 | 89 | 234-236 [238-240](Goli-Jolodar, Shirini et al. 2016) |
| 4 |   (**2d**) | **7d** | 45 | 92 | 196-198 [200-202](Gazerani, Malekzadeh et al. 2017) |
| 5 |   (**2e**) | **7e** | 45 | 84 | 182-184 [182-184](Zolfigol and Yarie 2015) |
| 6 |   (**2f**) | **7f** | 60 | 80 | 224-226 [226-228](Gazerani, Malekzadeh et al. 2017) |
| 7 |   (**2g**) | **7g** | 45 | 85 | 234-237 [238-241](Yarhosseini, Javanshir et al. 2016) |
| 8 |   (**2h**) | **7h** | 45 | 96 | 223-225 [224-226](Zolfigol and Yarie 2015) |
| 9 |   (**2i**) | **7i** | 55 | 96 | 235-237 [239-242](Gazerani, Malekzadeh et al. 2017) |
| 10 |   (**2j**) | **7j** | 25 | 93 | 260-263 [263-265](Zolfigol and Yarie 2015) |
| 11 |   (**2k**) | **7k** | 220 | 67 | 209-212 [208-211](Li, Zhong et al. 2015) |
| 12 |  (**2l**) | **7l** | 20 | 95 | 256-259 [255-257](Zolfigol and Yarie 2015) |
| 13 | (**2m**) | **7m** | 190 | 62 | 223-225 [225-227](Sakram, Sonyanaik et al. 2016) |
| 14 |   (**2n**) | **7n** | 80 | 70 | 230-232 [233-235](Tajbakhsh, Alinezhad et al. 2013) |
| 15 |   (**2o**) | **7o** | 90 | 65 | 186-188 [184-186](Tajbakhsh, Alinezhad et al. 2013) |
| 16 |   (**2p**) | **7p** | 120 | 74 | 218-221 [223-225](Tajbakhsh, Alinezhad et al. 2013) |
| 17 |   (**2q**) | **7q** | 90 | 56 | 203-20 [204-205](Yarhosseini, Javanshir et al. 2016) |
| 18 |   (**2r**) | **7r** | 90 | 67 | 166-168 [165-167](Davoodnia, Khashi et al. 2013) |
| 19 |   (**2t**) | **7t** | 55 | 94 | 273-275 [274-276](Yü, Wu et al. 2017) |
| 20 |   (**2u**) | **7u** | 45 | 80 | 157-160 [157-160](Ghorbani-Vaghei, Malaekehpoor et al. 2016) |
| ^a^Reaction conditions: aldehyde (**2**, 1 mmol), dimedone (**3**, 1 mmol), NH_4_OAc (**4**, 1.5 mmol) and ethyl acetoacetate (**5**, 1 mmol) in EtOH (2 ml); ^b^isolated yields. ^c^All products are known and their structures were established from their spectral data and melting points compared to authentic samples or literature values. | | | | | |

**Table** **4**. Comparative synthesis of compounds **5a** and **7a** using the reported methods versus the present method.

| **Entry** | **Catalyst** | **Product** | **Catalyst Loading (mg)** | **Solvent** | **T °C** | **Time (min.)** | **Yield** | **Ref** |
| --- | --- | --- | --- | --- | --- | --- | --- | --- |
| **1** | KH_2_PO_4_ | **5a** | 5 mol% | EtOH/H_2_O | 120 | 5 h | 94 | ^145^ |
| **2** | DABCO–PEG-400 ionic liquid | **5a** | 80 | - | 115 | 12-14 h | 92 | ^147^ |
| **3** | Silica bonded *N*-propyl sulfamic acid | **5a** | 30 | EtOH | Reflux | 2 h | 86 | ^148^ |
| **4** | Sawdust sulphonic acid | **5a** | 50 | EtOH | Reflux | 1 h | 90 | ^101^ |
| **5** | Fe_3_O_4_@SiO_2_@PTS-THEIC-(CH_2_)_3_OB(OH)_2_ | **5a** | 10 | EtOH | Reflux | 1 h | 92 | This Work |
| **6** | L-proline | **7a** | 10 | EtOH | Reflux | 360 | 92 | ^149^ |
| **7** | Yb(OTf)_3_ | **7a** | 60 | EtOH | 25 | 300 | 90 | ^150^ |
| **8** | PdRuNi@GO | **7a** | 6 | DMF | 70 | 45 | 92 | ^24^ |
| **9** | PS/PTSA | **7a** | 10 | - | 60 | 45 | 75 | ^151^ |
| **10** | Fe_3_O_4_@B-MCM-41 | **7a** | 50 | EtOH | Reflux | 40 | 92 | ^152^ |
| **11** | Silica Sulfuric Acid (SSA) | **7a** | 80 | - | 60 | 45 | 93 | ^153^ |
| **12** | PMO-ICS-PrSO_3_H | **7a** | 20 | EtOH | Reflux | 20 | 95 | ^118^ |
| **13** | Fe_3_O_4_@SiO_2_@PTS-THEIC-(CH_2_)_3_OB(OH)_2_ | **7a** | 10 | EtOH | Reflux | 20 | 95 | This Work |

# **9-(4-Chlorophenyl)-3,3,6,6-tetramethyl-3,4,6,7,9,10-hexahydro-1,8(2*H*,5*H*)-acridinedione (5a):**

Pale yellow solid; m.p.: 310-312 °C, FTIR (KBr, cm^-1^): 3282, 3176, 3060, 2954, 2875,1650, 1608, 1492, 1365, 1220, 1147, 1089, 1014, 840, 761, 597, 526; ^1^H NMR (500 MHz, CDCl_3_): δ (ppm): 0.98 (s, 6H, 2CH_3_), 1.10 (s, 6H, 2CH_3_), 2.19-2.37 (8H, m, 4CH_2_), 5.06 (s, 1H, CH), 7.17 (d, 2H, Ar-H), 7.28 (d, 2H, Ar-H), 6.97 (s, 1H, NH).

| 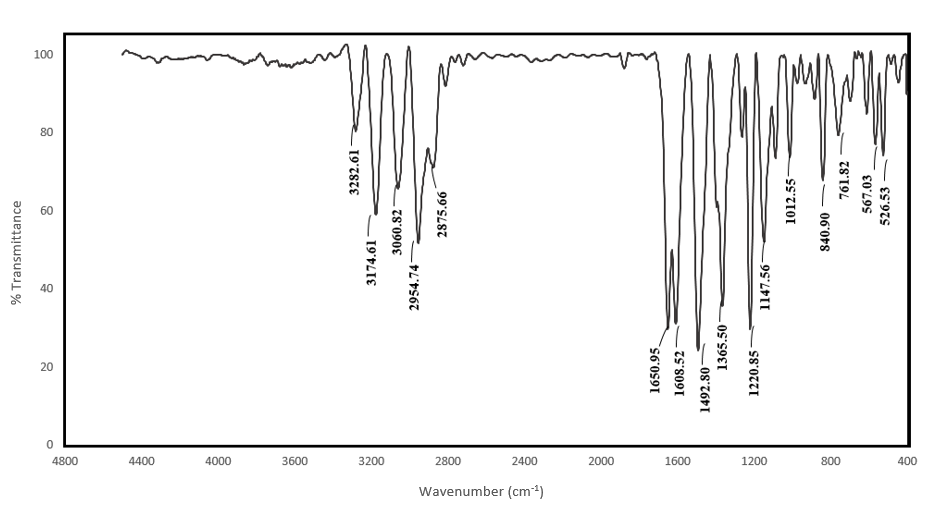 |
| --- |

**Fig. 10.** IR Spectrum of 9-(4-Chlorophenyl)-3,3,6,6-tetramethyl-3,4,6,7,9,10-hexahydro-1,8(2*H*,5*H*)-acridinedione (**5a**, reproduced using the Microsoft Excel 2016).

| 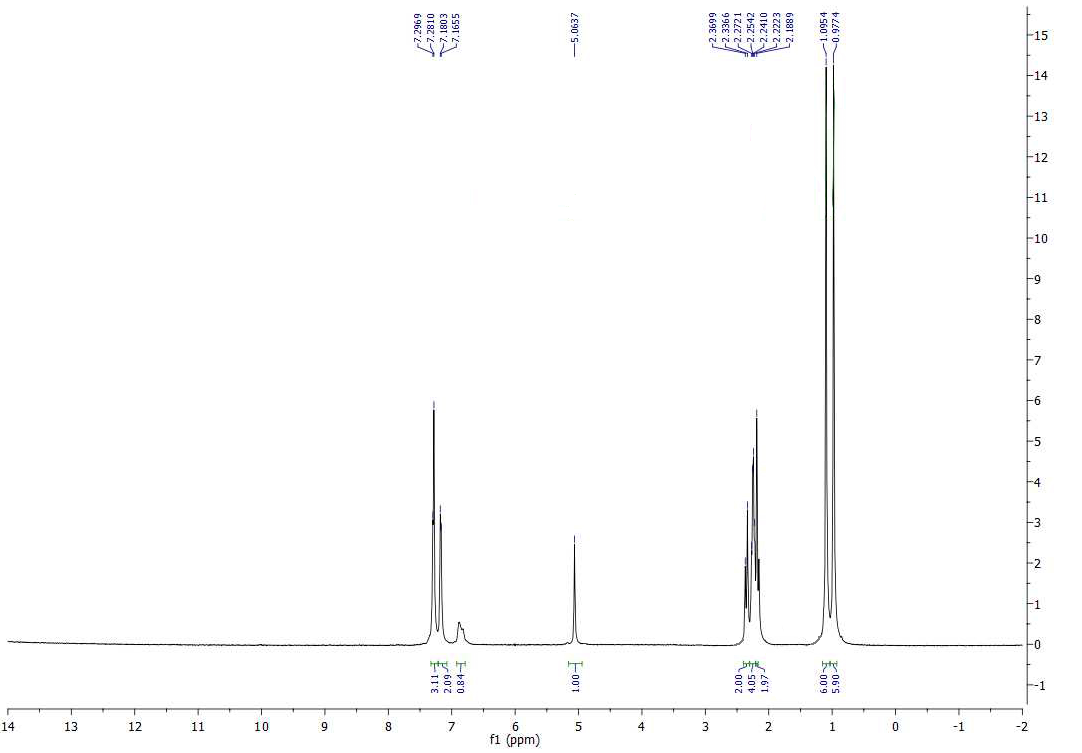 |
| --- |
| **Fig. 11.** ^1^H NMR Spectrum of 9-(4-Chlorophenyl)-3,3,6,6-tetramethyl-3,4,6,7,9,10-hexahydro-1,8(2*H*,5*H*)-acridinedione (**5a**). |

# **9-(4-Methoxyphenyl)-3,3,6,6-tetramethyl-3,4,6,7,9,10-hexahydro-1,8(2*H*,5*H*)-acridinedione (5d):**

Pale yellow solid; m.p.: 311-314 °C, FTIR (KBr, cm^-1^): 3276, 3205, 3068, 2954, 1641, 1479, 1365, 1224, 1141, 1031, 837, 565; ^1^H NMR (500 MHz, CDCl_3_): δ (ppm): 0.95 (s, 6H, 2CH_3_), 1.06 (s, 6H, 2CH_3_), 2.13-2.31 (8H, m, *J= 16,* 4CH_2_), 3.68 (s, 3H, OCH_3_), 5.02 (s, 1H, CH _benzylic_), 5.98 (s, 1H, NH), 6.70 (d, 2H, Ar-H), 7.23 (d, 2H, Ar-H)

| 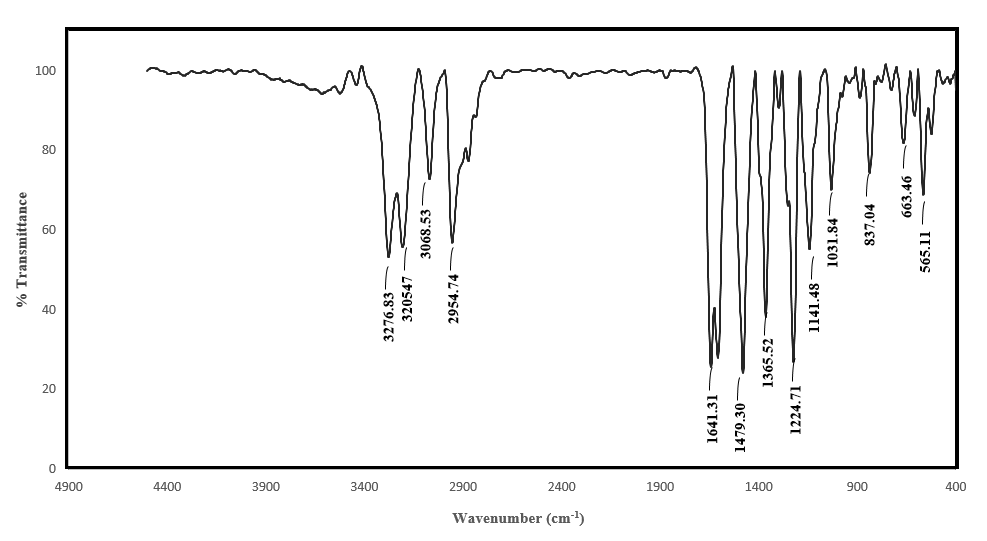 |
| --- |
| **Fig. 12.** IR Spectrum of 9-(4-Methoxyphenyl)-3,3,6,6-tetramethyl-3,4,6,7,9,10-hexahydro-1,8(2*H*,5*H*)-acridinedione (**5d**, reproduced using the Microsoft Excel 2016). |

| 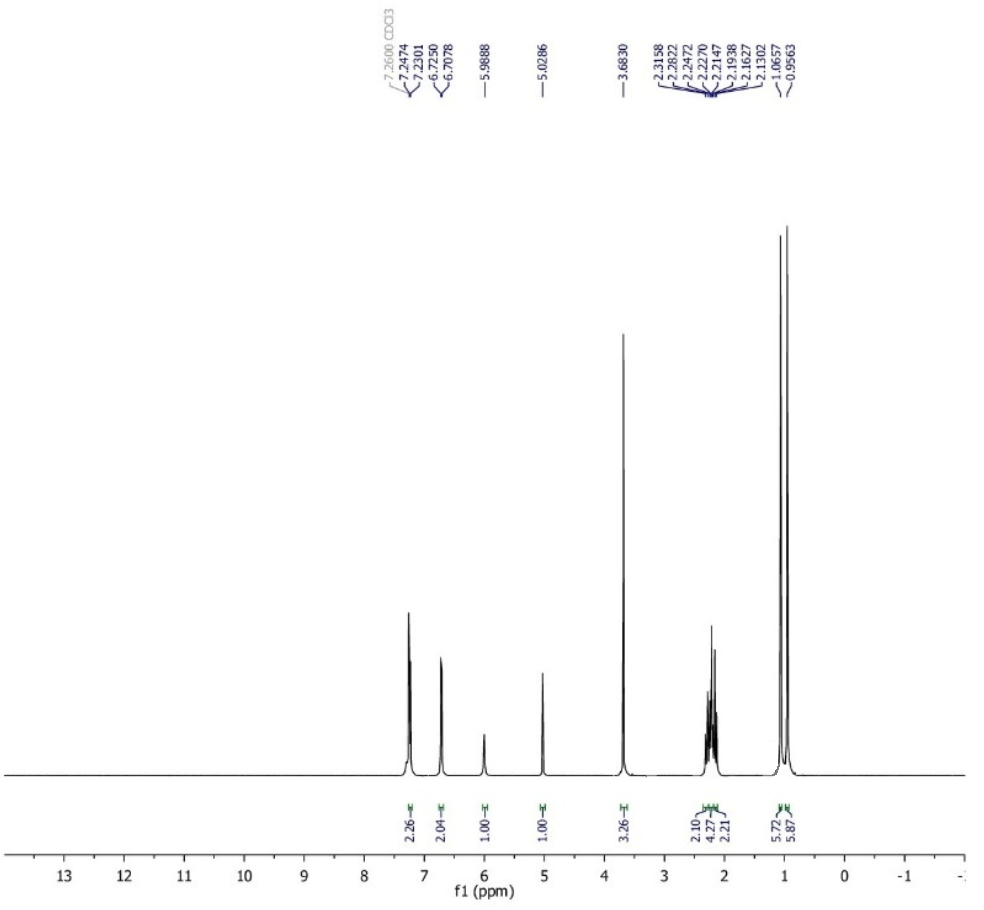 |
| --- |
| **Fig. 13.** ^1^H NMR Spectrum of 9-(4-Methoxyphenyl)-3,3,6,6-tetramethyl-3,4,6,7,9,10-hexahydro-1,8(2*H*,5*H*)-acridinedione (**5d**). |

**Ethyl 2,7,7-trimethyl-4-(3-nitrophenyl)-5-oxo-1,4,5,6,7,8-hexahydroquinoline-3-carboxylate (6e):**

Pale yellow solid; m.p.: 180-184 °C, FTIR (KBr, cm^-1^): 3276, 3193, 2964, 1703, 1604, 1490, 1379, 1278, 1215, 1143, 1070, 1022, 829, 754, 690, 507; ^1^H NMR (500 MHz, CDCl_3_): δ (ppm): 0.93 (s, 3H, CH_3_), 1.09 (s, 3H, CH_3_), 1.19 (t, 3H, *J= 7.15*, CH_3 (OEt)_), 2.13-2.40 (7H, s CH_3_, m 2CH_2_), 4.03-4.07 (q, 2H, *J= 7.15*, CH_2 (OEt)_), 5.15 (s, 1H, CH_benzylic_), 5.98 (s, 1H, NH), 7.35-8.10 (m, 2H, Ar-H)

| 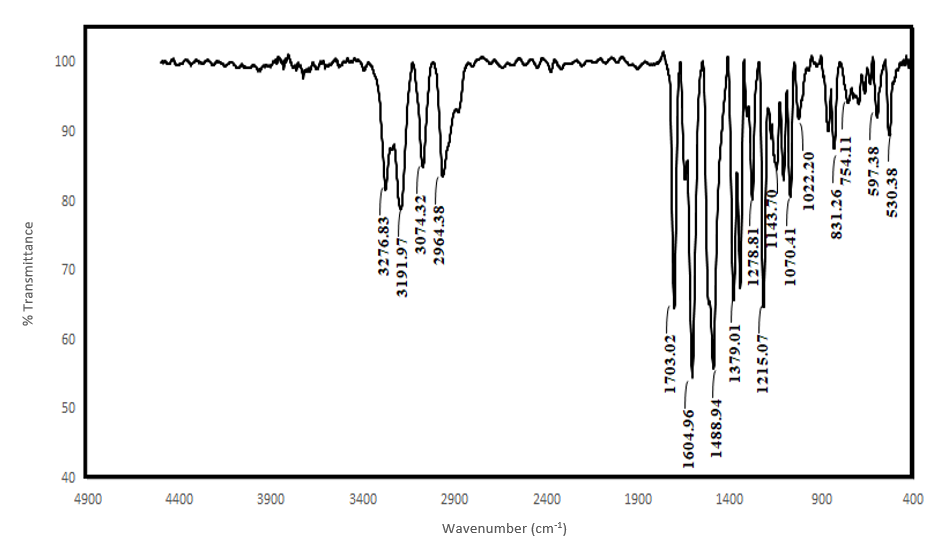 |
| --- |
| **Fig. 14.** IR Spectrum of ethyl 2,7,7-trimethyl-4-(3-nitrophenyl)-5-oxo-1,4,5,6,7,8-hexahydroquinoline-3-carboxylate (**6e**, reproduced using the Microsoft Excel 2016). |

| 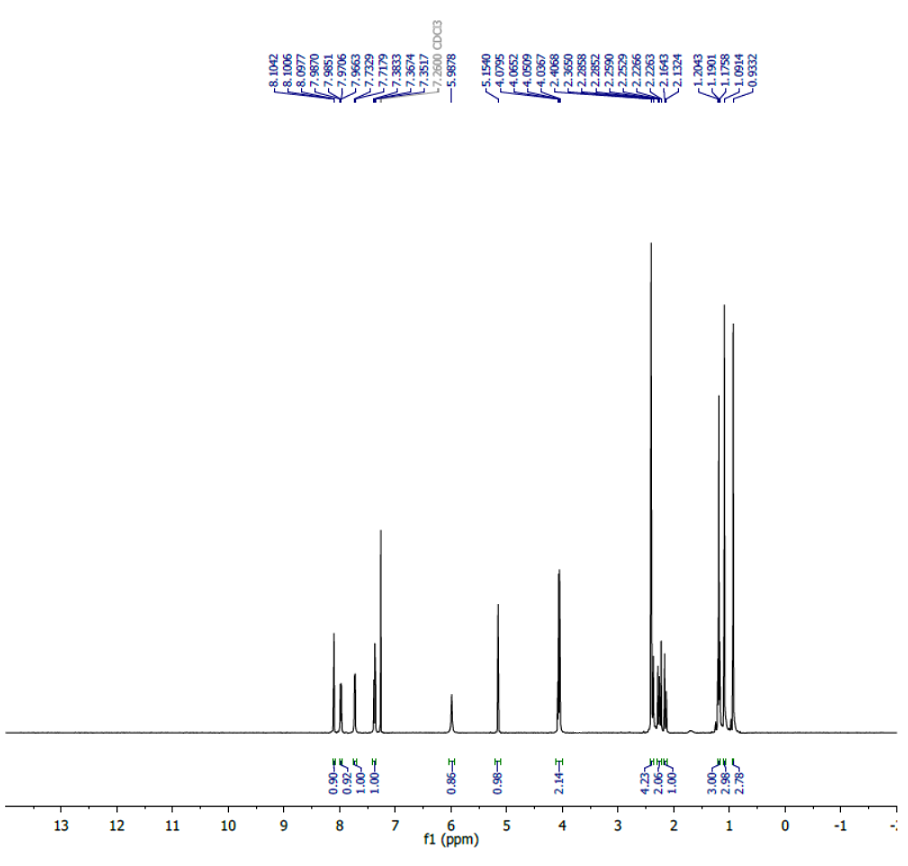 | |
| --- | --- |
| **Fig. 15.** ^1^H NMR Spectrum of ethyl 2,7,7-trimethyl-4-(3-nitrophenyl)-5-oxo-1,4,5,6,7,8-hexahydroquinoline-3-carboxylate (**6e**). | |

**Ethyl 4-(4-methoxyphenyl)-2,7,7-trimethyl-5-oxo-1,4,5,6,7,8-hexahydroquinoline-3-carboxylate (6l):**

Pale yellow solid; mp.: 255-260 °C, FTIR (KBr, cm^-1^): 3278, 3203, 3076, 2956, 1699, 1604, 1496, 1379, 1276, 1218, 1070, 1031, 842, 765, 536; ^1^H NMR (500 MHz, CDCl_3_): δ (ppm): 0.92 (s, 3H, CH_3_), 1.04 (s, 3H, CH_3_), 1.20 (t, 3H, *J= 7.15*, CH_3 (OEt)_), 2.11-2.28 (m, 4H, CH_2_), 2.33 (s, 3H, CH_3_), 3.71 (s, 3H, OCH_3_), 4.03-4.07 (q, 2H, *J= 7.15*, CH_2 (OEt)_), 4.98 (s, 1H, CH _benzylic_), 6.43 (br s, 1H, NH), 6.71-6.73 (d, 2H, *J=* 8.2, Ar-H), 7.21 (d, 2H, *J= 8.2*, Ar-H)

| 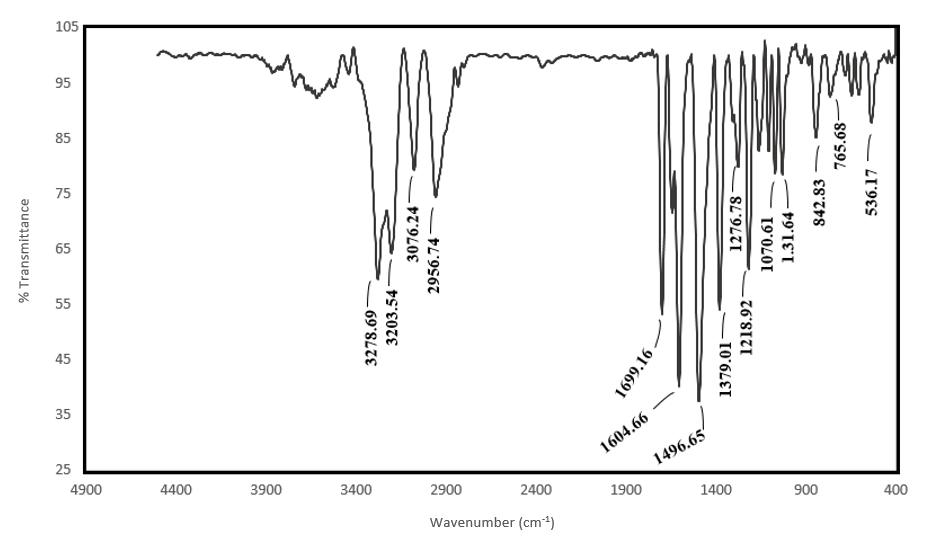 |
| --- |
| **Fig. 16.** IR Spectrum of ethyl 4-(4-methoxyphenyl)-2,7,7-trimethyl-5-oxo-1,4,5,6,7,8-hexahydroquinoline-3-carboxylate (**6l**, reproduced using the Microsoft Excel 2016). |

| 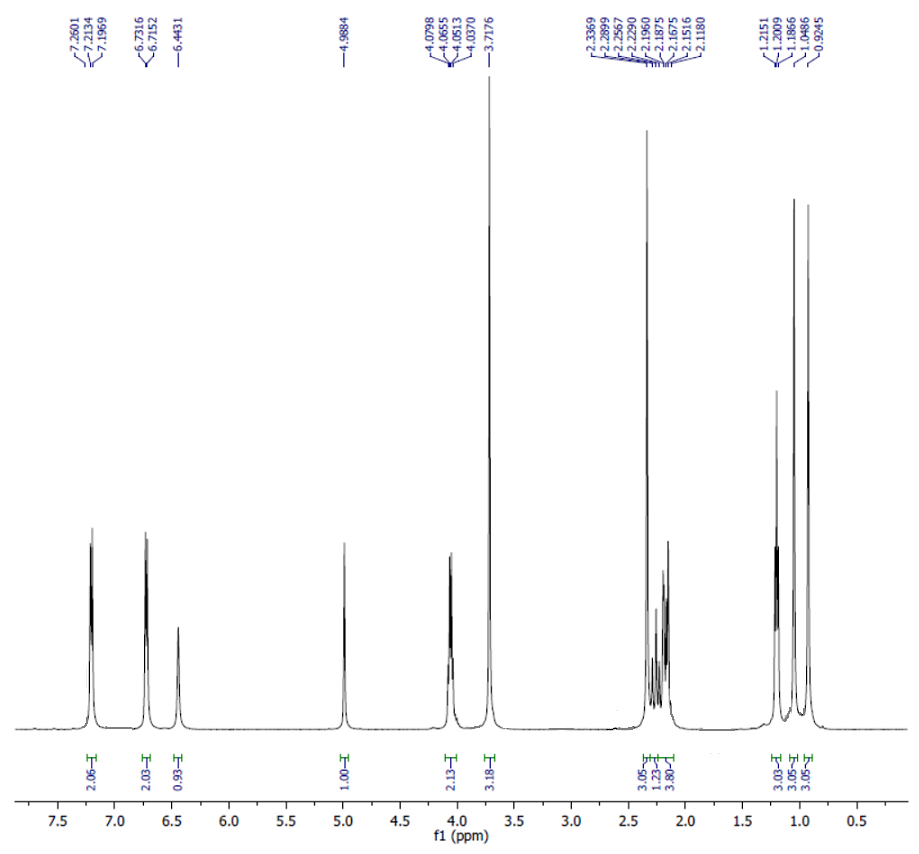 | |
| --- | --- |
| **Fig. 17.** ^1^H NMR Spectrum of ethyl 4-(4-methoxyphenyl)-2,7,7-trimethyl-5-oxo-1,4,5,6,7,8-hexahydroquinoline-3-carboxylate (**6l**). | |
